# Genoarchitecture of the extended amygdala in zebra finch, and expression of FoxP2 in cell corridors of different genetic profile

**DOI:** 10.1007/s00429-016-1229-6

**Published:** 2016-05-09

**Authors:** Alba Vicario, Ezequiel Mendoza, Antonio Abellán, Constance Scharff, Loreta Medina

**Affiliations:** 1 Laboratory of Brain Development and Evolution, Department of Experimental Medicine, Faculty of Medicine, University of Lleida, Institute of Biomedical Research of Lleida (IRBLleida), Avda. Alcalde Rovira Roure 80, Catalunya, 25198 Lleida, Spain; 2 Freie Universität Berlin, 14195 Berlin, Germany

**Keywords:** Intercalated amygdalar cells, Central amygdala, Medial amygdala, Bed nucleus of the stria terminalis, Mesotocin, FoxP2, Enkephalin, Somatostatin, Social behavior, Evolution, Striatal, Pallidal, Preoptic, Prethalamic eminence, Pallium, Nucleus taeniae

## Abstract

We used a battery of genes encoding transcription factors (Pax6, Islet1, Nkx2.1, Lhx6, Lhx5, Lhx9, FoxP2) and neuropeptides to study the extended amygdala in developing zebra finches. We identified different components of the central extended amygdala comparable to those found in mice and chickens, including the intercalated amygdalar cells, the central amygdala, and the lateral bed nucleus of the stria terminalis. Many cells likely originate in the dorsal striatal domain, ventral striatal domain, or the pallidal domain, as is the case in mice and chickens. Moreover, a cell subpopulation of the central extended amygdala appears to originate in the prethalamic eminence. As a general principle, these different cells with specific genetic profiles and embryonic origin form separate or partially intermingled cell corridors along the extended amygdala, which may be involved in different functional pathways. In addition, we identified the medial amygdala of the zebra finch. Like in the chickens and mice, it is located in the subpallium and is rich in cells of pallido-preoptic origin, containing minor subpopulations of immigrant cells from the ventral pallium, alar hypothalamus and prethalamic eminence. We also proposed that the medial bed nucleus of the stria terminalis is composed of several parallel cell corridors with different genetic profile and embryonic origin: preoptic, pallidal, hypothalamic, and prethalamic. Several of these cell corridors with distinct origin express FoxP2, a transcription factor implicated in synaptic plasticity. Our results pave the way for studies using zebra finches to understand the neural basis of social behavior, in which the extended amygdala is involved.

## Introduction

Zebra finches (*Taeniopygia guttata*; order Passeriformes) are a highly gregarious species of songbirds, that learn and use song for social communication (Riters et al. 2004; Fisher and Scharff 2009; Goodson 2013; Wohlgemuth et al. 2014), and are widely employed for social behavior studies (Goodson et al. 2009; Kelly et al. 2011; Goodson 2013; Kelly and Goodson 2013, 2014; Kingsbury and Goodson 2014). The extended amygdala is highly relevant for controlling or modulating this behavior [reviewed by Martínez-García et al. (2007) and Abellán et al. (2013)], but its structure is poorly defined in songbirds. The extended amygdala consists of two major parts: the medial extended amygdala (EAme, including the medial amygdala and medial bed nucleus of the stria terminalis or BSTM) and the central extended amygdala (EAce, including the central amygdala, the intercalated amygdalar cells and the lateral bed nucleus of the stria terminalis or BSTL) (Alheid and Heimer 1988; de Olmos et al. 2004). In mammals, the EAme is particularly relevant for aspects of social behavior related to affiliation, agonistic behavior (including aggresion/defense) and sexual behavior (Choi et al. 2005; Hammock and Young 2006), while the EAce is essential for fear/anxiety responses and reward (Davis 1992; Walker et al. 2003; Kalin et al. 2004; Phelps and LeDoux 2005; Walker and Davis 2008; Walker et al. 2009; Davis et al. 2010; reviewed by Martínez-García et al. 2007, 2012), and is also relevant for modulating some of the emotional aspects that drive social behavior (Moore and Isen 1990). Both parts of the extended amygdala have been recently redefined in mice and chickens based on expression of transcription factors during development and the embryonic origin of their neurons (mouse: García-López et al. 2008; Bupesh et al. 2011a, b; chicken: Abellán and Medina 2009; Abellán et al. 2013; Vicario et al. 2014, 2015). Multiple embryonic domains produce neurons for the EAce and EAme. Neurons produced in each distinct domain are characterized by specific genetic profiles and distribute, by way of radial or tangential migrations, along the extended amygdala, forming corridors of cells with a similar phenotype that are apparently enrolled in a similar functional pathway (reviewed by Abellán et al. 2013 for the EAme; see Bupesh et al. 2011b, and Vicario et al. 2014, 2015, for the EAce). These data open a new venue for trying to understand the functional organization of the extended amygdala, and the multifaceted modulation of social behavior by this complex structure.

Although the different cellular components of the EAme and EAce have been identified in chickens and could thus be compared to those in mice (Abellán and Medina 2009; Vicario et al. 2014, 2015), these data are not easily translatable to zebra finches or other songbirds due to the high evolutionary divergence between Galliformes and Passerifomes (Jarvis et al. 2014), especially affecting the telencephalic hemispheres (Iwaniuk and Hurd 2005). Studies on the neural basis of social behavior in zebra finches specifically mention the medial amygdala and the BSTM (for example, Goodson et al. 2012; Kelly and Goodson 2013). However, the pallial or subpallial nature of the putative homolog of the mammalian medial amygdala in zebra finch (the so-called nucleus taeniae, Ikebuchi et al. 2013) is unclear. This is an important issue, since the medial amygdala in other vertebrates (including chicken) is primarily a subpallial nucleus rich in neurons of pallidal and preoptic origins, although it also includes some minor subpopulations of immigrant neurons coming from the ventral pallium or from the hypothalamus (García-López et al. 2008; Abellán and Medina 2009; Bupesh et al. 2011a). As the medial amygdala, the BSTM is known to include different neuron subpopulations derived from the pallidum, preoptic area, hypothalamus and possibly also from the prethalamic eminence (García-López et al. 2008; Abellán and Medina 2009; Bupesh et al. 2011a). Each of these different cell types may belong to a different functional pathway, being able to modulate or control a different aspect of behavior (Medina et al. 2011; Abellán et al. 2013). However, this is unexplored in zebra finches. In addition, the central extended amygdala, involved in fear/anxiety responses and reward (Martínez-García et al. 2007), has not been studied in zebra finches or other songbirds at all. A recent study used zebra finches as a model for analyzing the expression of a battery of developmental regulatory genes during development, which has been useful for a better delineation of pallial and subpallial structures, and their subdivisions (Chen et al. 2013). The expression of some transcription factors in the zebra finch in that study corroborated previous findings in the chicken (Puelles et al. 2000; Abellán and Medina 2009; Abellán et al. 2009), but the extended amygdala was not analyzed. Given the relevance of this structure for fully understanding the neural basis of social behavior, we undertook a thorough analysis of this region in the zebra finch from late embryonic through early posthatching development to juvenile stages, using a battery of transcription factors (such as Pax6, Islet1, Nkx2.1, Lhx6, Lhx5 and Lhx9) and other proteins (such as proenkephalin, somatostatin and mesotocin) useful for delineating different components of the extended amygdala in mice and chickens. In addition, we investigated the expression of FoxP2 in the different components of the extended amygdala, since alterations in the gene encoding this transcription factor have been associated with language learning deficits in humans (Lai et al. 2001; Haesler et al 2007; reviewed by Fisher and Scharff 2009, Fischer and Hammerschmidt 2011, and French and Fisher 2014), and may contribute (not alone, but combination with other genes) to the development of autism (Park et al. 2014), which implies not only deficits in communication but also in social skills (Bacon and Rappold 2012). Songbirds like the zebra finch are excellent models for studying the role of FoxP2 in the brain, since learned songs are used for social communication, and this transcription factor is regulated by singing and in fact required for proper song learning and song maintenance (Scharff and Haesler 2005; Wohlgemuth et al. 2014; Murugan et al. 2013; Condro and White 2014; Heston and White 2015). In mice, FoxP2 is expressed in the extended amygdala, including the intercalated amygdalar cells and the medial amygdala (Campbell et al. 2009; Kaoru et al. 2010), but, other than that, the exact location of the expression within this mosaic-like complex structure is unknown. We thus used the zebra finch to map FoxP2 expression in cell components of the EAce and EAme with different embryonic origins and genetic profiles.

## Materials and methods

In the present study, we used domestic zebra finch (*Taeniopygia guttata*) embryos from embryonic day 14 (E14; St. 44, following the embryonic stages classification of Murray et al. 2013) until post-hatching day 50 (PHD50). The correspondence between the embryonic days of the finches used in this study and the stages proposed by Murray et al. (2013) is close but not identical. This is likely the result of differences in incubation: Murray et al. (2013) used an incubator, while we collected eggs in an outside aviary. The latter develop slower, possibly due to the temperature variations. All animals were treated according to the regulations and laws of the European Union (Directive 2010/63/EU) and in accordance with regulations established by the Landesamt für Gesundheit und Soziales of Berlin for care and handling of animals in research. The protocols used were approved by the afore-mentioned committee. For the embryo extraction, we followed a modification of the protocol described by Murray et al. (2013). Embryos were first placed on ice to reduce body temperature and induce analgesia. Then, they were rapidly decapitated and their heads were fixed by immersion in phosphate-buffered 4 % paraformaldehyde (pH 10.5, to preserve mRNA integrity, Basyuk et al. 2000). The hatched individuals received an overdose of isofluorane (Baxter Healthcare Corporation) prior to sacrifice. The brains were dissected and fixed in the same way as described above for the embryos. Juveniles (from PHD11-PHD25) were overdosed with isofluorane and subsequently perfused transcardially with the same fixative solution and, following dissection, the brains were postfixed for 24 h at 4 °C. After postfixation, brains were embedded in 4 % low-melt agarose and sectioned (70–90 µm-thick) in frontal or sagittal planes using a vibratome (Leica VT 1000S). Brain sections were then processed for in situ hybridization or/and immunofluorescence (Table 1).

**Table 1:** Brains processed per each gene product using in situ hybridization and/or immunofluorescence

Gene product	Cases analyzed
cIslet1	7
cLhx6	6
cLhx9	5
cNkx2.1	7
cPax6	11
cpENK	7
cSOM	3
cLhx5	8
zMes	5
FoxP2	19

## In situ hybridization

Frontal or sagittal brain sections were processed for in situ hybridization using digoxigenin-labeled riboprobes, following a procedure previously described (Medina et al. 2004; García-López et al. 2008; Abellán and Medina 2009). The riboprobes were synthesized from cDNAs of different genes (mostly from chicken, except two, as explained below), which were purchased, obtained from other laboratories, or cloned. The purchased chicken clones were cDNA ESTs obtained from the BBSRC ChickEST Database [Boardman et al. 2002; purchased from ARK-genomics (Roslin Institute; Midlothian, UK) or Geneservice Limited (Cambridge, UK)], and have a corresponding Genbank accesssion number. Before using the riboprobes of chicken gene fragments for the in situ hybridization in zebra finch, we first checked the homologies between the chicken probe sequence and the zebra finch genes. This was feasible because the zebra finch genome is completely sequenced (Warren et al. 2010; http://www.ncbi.nlm.nih.gov/projects/genome/guide/finch/). Chicken probe information and percentage of homology with zebra finch are given below and in Table 2:

**Table 2:** Information on the genes used for preparing the riboprobes

Gene	Base pairs	Genbank accession number	BBSRC code	Probe homology (%)	Gene homology (%)	Zebra Finch chromosome
cIslet1	1–452	NM_205414.1	ChEST314A21	91.8	94.9	Z chromosome
cLhx6	1–698	DQ082894.1	ChEST365j8	94.9	94	Chromosome 17
cLhx9	1–613	NM_205426	ChEST664o12	95.7	97	Chromosome 8
cNkx2.1	1–1125	AF110995	Not applicable	93.6	92	Chromosome 5
cPax6	849–1964	NM_205066.1	Not applicable	95.8	93	Chromosome 5
cpENK	3–865	XM_419213.3	ChEST140a9	90.6	90.6	Chromosome 2
cSST	40–707	NM_205336.1	ChEST114E9	93	90	Chromosome 9
zLhx5	1–923	Not applicable	Not applicable	100	100	Chromosome 15
zMes	36–358	Not applicable	Not applicable	100	100	Chromosome 4

- cIslet1 (bp 1–452; Genbank accession no: NM_205414.1; BBSRC ChickEST Database; clone ChEST314A21). Homology percentage of chicken probe with zebra finch gene: 91.8 % (aligned to a sequence in Z chromosome of zebra finch with a 91.8 % of homology, corresponding to 6–352 bp of the chicken riboprobe). The complete chicken gene has 94.9 % of homology compared to the zebra finch gene.
- cLhx6 (bp 1–698; Genbank accession no: DQ082894.1; BBSRC ChickEST Database: clone ChEST365j8). Homology percentage of chicken probe with zebra finch gene: 94.9 % (aligned to a sequence in chromosome 17 with a 94.9 % of homology, corresponding to 14–667 bp of the chicken riboprobe). The complete chicken gene has 94 % of homology compared to the zebra finch gene.
- cLhx9 (bp 1–613; Genbank accession no: NM_205426; BBSRC ChickEST Database: clone ChEST664o12). Homology percentage of chicken probe with zebra finch gene: 95.7 % (aligned to a sequence in chromosome 8 with a 95.7 % of homology, corresponding to 1–749 bp of the chicken riboprobe). The complete chicken gene has 97 % of homology compared to the zebra finch gene.
- cNkx2.1 (bp 1–1125; Genbank accession no: AF110995; plasmid obtained from J.L.R. Rubenstein's lab; Puelles et al. 2000). Homology percentage of chicken probe with zebra finch gene: 93.6 % (aligned to a sequence in chromosome 5 with a 93.6 % of homology, corresponding to 1–1125 bp of the chicken riboprobe). The complete chicken gene has 92 % of homology compared to the zebra finch gene.
- cPax6 (bp 849–1964; Genbank accession no: NM_205066.1; plasmid obtained from J.L.R. Rubenstein’s lab; Puelles et al. 2000). Homology percentage of chicken probe with zebra finch gene: 95.8 % (aligned to a sequence in chromosome 5 with a 95.8 % of homology, corresponding to 349–1960 bp of the chicken riboprobe). The complete chicken gene has 93 % of homology compared to the zebra finch gene.
- pro-enkephalin (pENK; bp 3–865; Genbank accession no: XM_419213.3; BBSRC ChickEST Database; clone ChEST140a9). Homology percentage of chicken probe with zebra finch gene: 90.6 % (aligned to a sequence in chromosome 2 with a 90.6 % of homology, corresponding to 4–811 bp of the chicken riboprobe). The complete chicken gene has 90.5 % of homology compared to the zebra finch gene.
- somatostatin precursor (SOM or SST; bp 40–707; Genbank accession no: NM_205336.1; BBSRC ChickEST Database; clone ChEST114E9). Homology percentage of chicken probe with zebra finch gene: 93 % (aligned to a sequence in chromosome 9 with a 93 % of homology, corresponding to 77–707 bp of the chicken riboprobe). The complete chicken gene has 90 % of homology compared to the zebra finch gene.

We synthesized the antisense digoxigenin-labeled riboprobes using Roche Diagnostics´s (Mannheim, Germany) protocols for the genes mentioned above.

To obtain zebra finch Lhx5 and Mesotocin we blasted the corresponding chicken sequences against the zebra finch data base (http://blast.ncbi.nlm.nih.gov/Blast.cgi) and used the Vector NTI program to construct the predicted zebra finch “Lhx5” and “Mesotocin”sequence. We designed primers to amplify part of the coding region of zebra finch “Lhx5” and “Mesotocin” (Mes). Primers were as follows: forward zLhx5: TTCTCCAGGGAAGGGAAACT; reverse zLhx5: CTAAGCGGACACCACTCCTC; forward zMes: CTCTCCTCCGCTTGCTACAT; reverse zMes: TGACCAGGAGATGCTGTTTG. The resulting PCR products (923 base pairs for zLhx5 and 378 base pairs for zMes) were examined on a TAE agarose gel, cleaned from nucleotides with the Nucleo spin purification kit (Machenery-Nagel, Germany), and cloned into pGEMTeasy vector (Promega, Madison, WI). Inserts from three independent “zLhx5” and “zMes” clones were then sequenced on both strands. Consensus sequence was built using the Vector NTI program. The cloned fragments encompass the sequence spanning from positions bp 1 to 923 of zLhx5 and bp 36 to 358 of zMes. The probes were generated from PCR-amplified sequences using M13 primers and T7 or SP6 RNA polymerase to drive the transcription of the mRNA sense and anti-sense probes.

Before hybridization, the sections were washed in PBS containing 0.1 % Tween-20 (PBT 1X), prehybridized in hybridization buffer (HB) for 2 h at 58 °C (for the post-hatching individuals) or 65 °C (for the embryos cases), and then hybridized in HB containing the riboprobe overnight at 58 or 65 °C, as explained before (0.5–1 µg/ml, depending on the probe and brain size). The hybridization buffer contained 50 % of deionized formamide, 1.3X standard saline citrate (SSC; pH 5), 5 mM ethylene-diamine-tetraacetic acid (EDTA; pH 8.0; Sigma-Aldrich, Steinheim, Germany), 1 mg/ml of yeast tRNA (Sigma-Aldrich), 0.2 % Tween-20, 100 µg/ml of heparin (Sigma-Aldrich), completed with water (free of RNAase and DNAase; Sigma-Aldrich). Following hybridization, the sections were washed with a mix 1:1 of MABT 1X (1.2 % maleic acid, 0.8 % NaOH, 0.84 % NaCl and 0.1 % Tween-20) and HB at 58 or 65 °C during 20 min and washed abundantly at room temperature with MABT 1X (about 2 h). Following this, the sections were blocked with a solution containing blocking reagent (Roche), MABT 1X and sheep serum (Sigma) for 4 h at room temperature, then incubated in an antibody against digoxigenin (alkaline-phosphatase coupled anti-digoxigenin; diluted 1:3500; Roche Diagnostics) overnight at 4 °C, later washed with MABT 1X and finally revealed with BM purple (Roche Diagnostics). Sections were then mounted on glycerol gelatine (Keisers Glycerol) or were processed for immunofluorescence (as explained in next section) and then mounted on immuMount (Thermo Scientific). This mounting media is suitable for the immunofluorescence cases, but not for the in situ hybridization, as it decays the signal.

## Immunofluorescence

Alternative series of sections and some previously hybridized sections were processed for immunofluorescence to detect FoxP2 (IgG polyclonal goat anti-Foxp2; AbCam, ab1307; against the synthetic peptide REIEEEPLSEDLE, corresponding to C terminal amino acids 703–715 of Human FOXP2). The specificity of this antibody has been checked in zebra finch brain by Western blot and by preincubating the antibody with the FoxP2 protein prior the immunohistochemistry (Thompson et al. 2013). With Western blot, FoxP2 labels a single band of approximately 80 kDa (Thompson et al. 2013; Mendoza et al. 2015). Briefly, floating brain sections were washed in 0.1 M phosphate buffered saline (PBS) containing 0.1 % Triton X-100 (PBS-TX, pH 7.4) for 15 min six times, and then blocked for 1 h in blocking buffer containing 0.4 % TX-100, 3 % bovine serum albumin, 5 % normal horse serum, and 0.1 % sodium azide in PBS. Afterwards, we incubated the sections overnight with a primary antibody against FoxP2 (1:2000) in blocking buffer in 4 °C. Following this incubation and standard washes in PBS-Triton, the sections were incubated in a secondary antiserum for 2 h at room temperature. The secondary antiserum used was rabbit anti-goat conjugated to Alexa 488 (Molecular Probes) and diluted 1:500. After incubation, the sections were rinsed and stored (at 4 °C, in the darkness) until they were analyzed with a fluorescence microscope.

## Image capture, manipulation, and figure assembly

Digital photographs of hybridized sections were obtained with a digital camera DC500 or DC350 (Leica, Wetzlar, Germany). Selected hybridized sections were re-taken on a Leica microscope (DMR HC) equipped with a Zeiss Axiovision digital camera. For fluorescence image acquisition, a Zeiss Axiovert S 100 microscope equipped with a Zeiss AxioCam HRc camera was used in a first approach. Later, selected sections processed for FoxP2 immunofluorescence and/or in situ hybridization for other transcription factors were analyzed and photographed using an Olympus BX51 microscope equipped for fluorescence and a digital camera (Olympus DP70), at Dr. Agustín González’s lab (University Complutense of Madrid). Selected digital images to be used for the figures were adjusted for brightness/contrast using Adobe PhotoShop and figures were prepared and labeled using FreeHand.

## Identification of cell masses and nomenclature

For identification of forebrain cell masses during development, we used the atlas of developing chicken brain (Puelles et al. 2007), as well as our own publications focussed on the amygdala (Abellán and Medina 2009; Abellán et al. 2010; Vicario et al. 2014). For a better comprehension of the similarities and differences between chicken and zebra finches brains, we also employed the Stereotaxic Atlas of The Brain of the Zebra Finch (Nixdorf-Bergweiler and Bishop 2007), the internet database http://www.zebrafinchatlas.org/, and The Zebrafinch Brain Architecture Project (http://zebrafinch.brainarchitecture.org/introduction/).

## Results

In this work, we present data on the mRNA expression of transcription factors and phenotypic markers that help to delineate different components of the extended amygdala of the zebra finch based on their apparent embryonic origin (given by the combinatorial expression of transcription factors analyzed during development within the brain topological framework) and mature neurochemical features (expression of different neuropeptides). The genes selected for this study include many of those previously used in chicken (Abellán and Medina 2009; Vicario et al. 2014) and mice (García-López et al. 2008; Abellán et al. 2010; Waclaw et al. 2010; Bupesh et al. 2011a, b), which were found to be relevant for identifying different cell subpopulations of the central and medial extended amygdala.

**Fig. 1:**
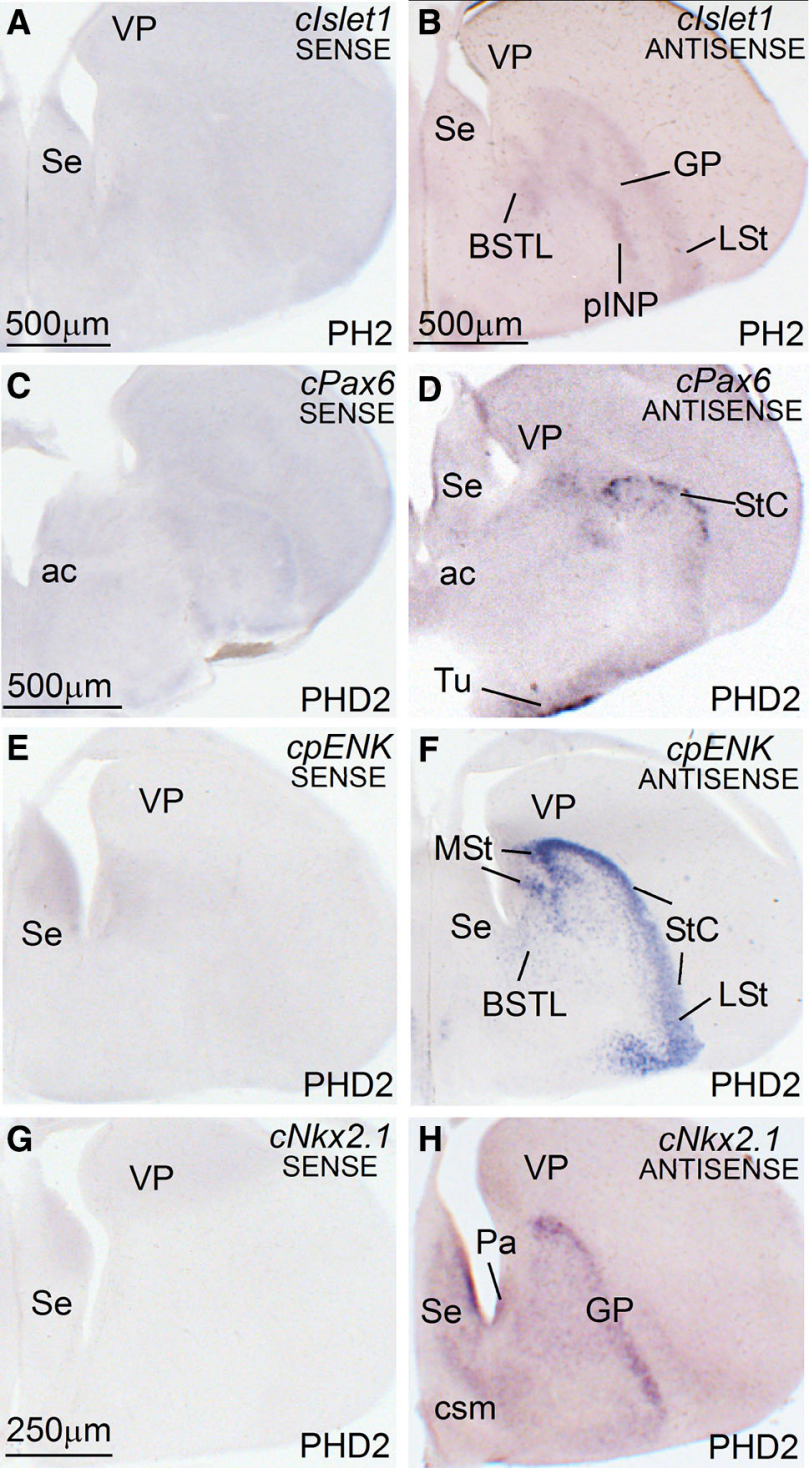
In situ hybridization of sense and antisense riboprobes from several chicken genes in the telencephalon of a 2-day-old posthatch (PHD2) zebra finch specimen. **a**–**h** Low-magnification digital images of parallel frontal telencephalic sections of a PHD2 zebra finch specimen, hybridized for sense (**a**, **c**, **e**, **g**) or antisense (**b**, **d**, **f**, **h**) riboprobes from the following chicken genes: cIslet1 (**a**, **b**), cPax6 (**c**, **d**), cpENK (**e**, **f**), and cNkx2.1 (**g**–**h**). All of the cases using antisense riboprobes showed expression patterns almost identical to those observed in chicken (See Vicario et al. 2014). For example, at the level of the sections shown in this figure (middle to caudal telencephalic levels, where parts of the basal ganglia and extended amygdala are seen), cIslet1 is expressed in derivatives of the ventral striatal division, including most of the medial and lateral striatum (MSt, LSt) and parts of the central extended amygdala (such as pINP and a subpopulation in BSTL) (**b**); cPax6 is expressed in derivatives of the dorsal striatal division, including the striatal capsule (StC) and part of the olfactory tubercle (Tu) (**d**); cpENK is strongly expressed in several striatal derivatives, such as MSt, LSt, and StC (**f**); and cNkx2.1 is expressed in pallidal structures, such as the globus pallidus (GP) and BSTL (**h**). In contrast, no signal was observed when using the sense riboprobe (**a**, **c**, **e**, **g**). For abbreviations, see list. *Scale bars* *A* = 500 μm; *B* = 500 μm; *C* = 500 μm (applies to **c**–**f**); *G* = 250 μm (applies to **g**–**h**)

Since for some of the transcription factors and phenotypic markers we used RNA-probes from chicken genes, we first carried out control experiments by hybridizing either the sense or the antisense riboprobes in parallel brain sections of zebra finch. We only used chicken genes in cases of sequence identity with the zebra finch genes above 90 % (see “Materials and methods”; Table 2), and these included Islet1, Pax6, pENK, and SOM (SST) for the central extended amygdala (EAce), and Nkx2.1, Lhx6, and Lhx9 for the medial extended amygdala (EAme). For all of these genes, the expression patterns visualized when using the antisense riboprobe in zebra finch brains were both consistent among animals and identical to those seen in chicken (Abellán and Medina 2009; Vicario et al. 2014, 2015). In contrast, no signal was observed when using the sense riboprobe. Examples of sense versus antisense hybridizations are shown in zebra finch brain sections, at the level of the extended amygdala, for cIslet1 (Fig. 1a, b), cPax6 (Fig. 1c, d), cpENK (Fig. 1e, f), and cNkx2.1 (Fig. 1g, h).

**Fig. 2:**
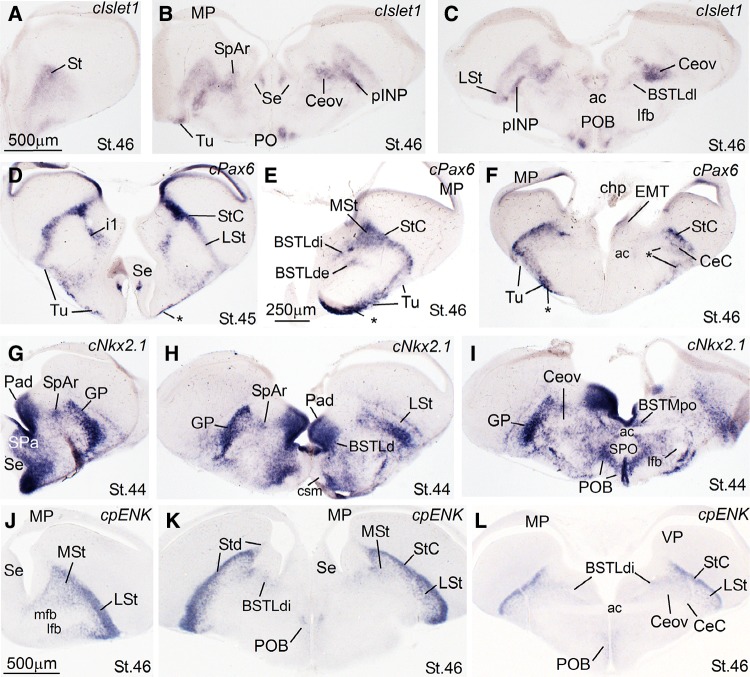
Expression of cIslet1, cPax6, cNKx2.1 and cpENK in the telencephalon of zebra finch embryos at pre-hatching stages (St. 44–St. 46). (**a**–**l**) Low-magnification digital images of frontal telencephalic sections of zebra finch embryos hybridized for cIslet1 (**a**–**c**), cPax6 (**d**–**f**), cNkx2.1 (**g**–**i**), and cpENK (**j**–**l**). For each gene, selected sections at rostral (**a**, **d**, **g**, **j**), intermediate (**b**, **e**, **h**, **k**), and caudal, commissural levels (**c**, **f**, **i**, **l**) of the zebra finch telencephalon are shown. The expression pattern of all the genes analyzed is similar to that observed in chicken. cIslet1 is strongly expressed in ventral striatal derivatives, including parts of the central extended amygdala (such as the pINP and Ceov). Subpopulations of cells are also seen in the SpAr and BSTLdl (**b**, **c**). In contrast, cPax6 is expressed in derivatives of the dorsal striatal subdivision (Std), including the striatal capsule (StC) and the capsular central amygdala (CeC). i1 in **d** points to a tangentially oriented cell corridor, expressing cPax6, extending from Std towards more ventral areas of the subpallium (see text for more details). The *asterisk* in **d**, **e** and **f** is showing an extratelencephalic input of cPax6-expressing cells, probably coming from the prethalamic eminence. cNkx2.1 is strongly expressed in pallidal and preoptic structures, as shown in (**g**–**i**). The pallidal domain in zebra finch seems to be bigger (protrudes more into the ventricle, resembling the medial ganglionic eminence) than in chicken (**h**). Note that the dorsal BSTL is adjacent to the vz/svz of the dorsal pallidal division (Pad) and contains many cells expressing cNkx2.1. As in chicken, cpENK is strongly expressed in striatal derivatives of zebra finches. The CeC and BSTLd also contain cells expressing enkephalin, but the signal in these nuclei seems to be more discrete in zebra finch than in chicken at prehatching stages, although later the signal intensifies (see Fig. 3i). In contrast, the signal for cIslet, cPax6 and cNkx2.1 is stronger at prehatching stages, but declines soon after hatching. For abbreviations, see list. *Scale bars* *A* = 500 μm (applies to **a**–**d**, **f**–**i**); *E* = 250 μm; *J* = 500 μm (applies to **j**–**l**)

**Fig. 3:**
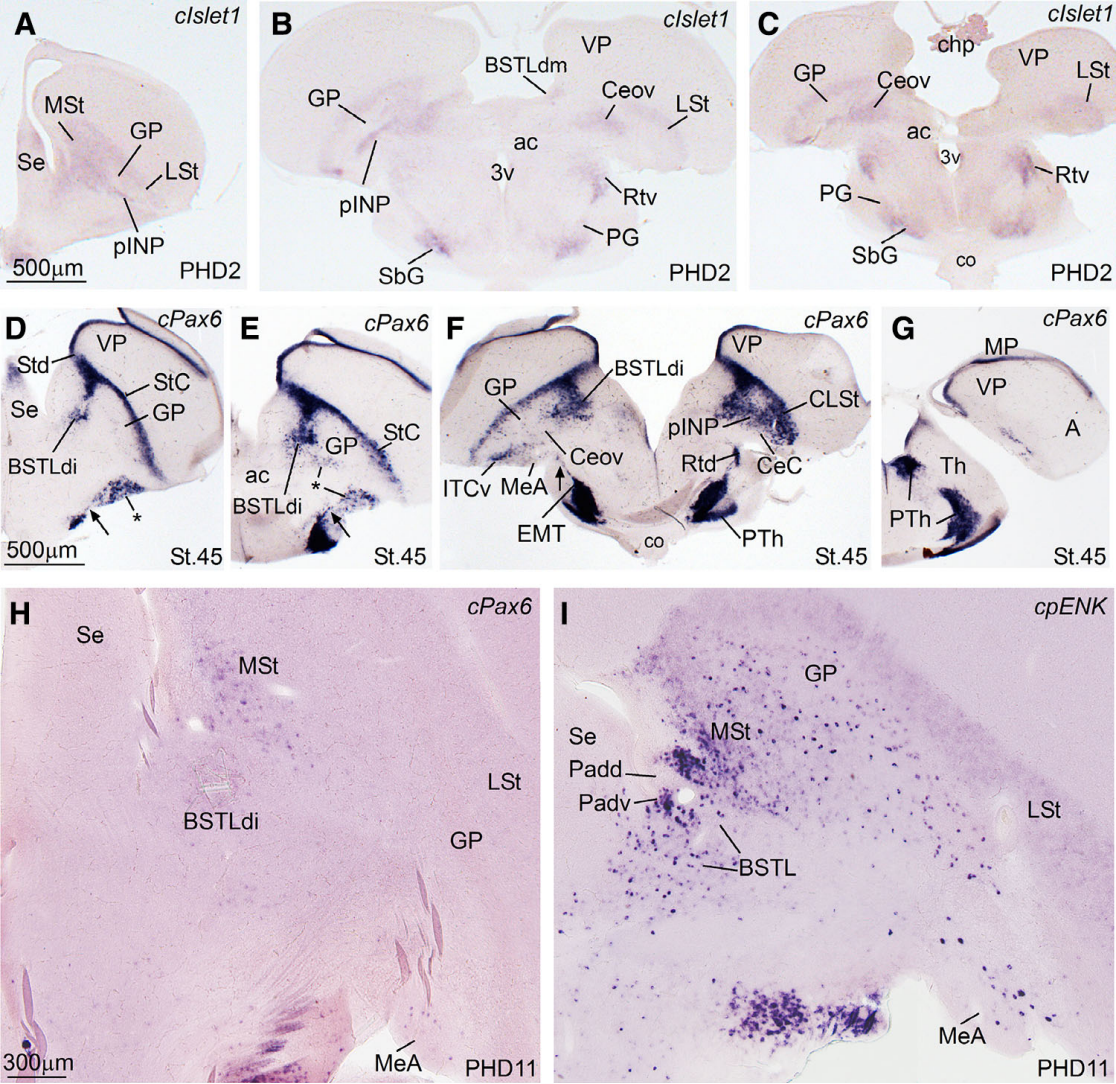
Expression of cIslet1, cPax6, and cpENK in the telencephalon of the zebra finch embryo at pre-hatching stages (St. 45), post-hatching stages (PHD2) and juveniles (PHD11). **a**–**g** Low-magnification digital images of oblique-horizontal telencephalic sections of a PHD2 zebra finch hybridized for cIslet1 (**a**–**c**), and a zebra finch embryo hybridized for cPax6 (**d**–**g**). **h**–**i** High-magnification digital images of frontal telencephalic sections of a PHD11 zebra finch hybridized for cPax6 **h**, and for cpENK **i**. cIslet1 is still expressed at PHD2 in the same striatal areas seen in embryos, including the pINP and Ceov of the central extended amygdala (EAce) seen in **a**–**c**. In addition note the moderate expression in the prethalamus. However, cIslet1 signal declines rapidly after hatching, and at PHD2 is rather weak in most of the striatal derivatives. cPax6 is strongly expressed in dorsal striatal derivatives, such as the dorsal and ventral intercalated-like cells (StC, ITCv), and the capsular central amygdala (CeC). Large subpopulations of cPax6 expressing cells also invade, apparently by tangential migration, the pINP and BSTLd. The *arrows* in **d**, **e** and **f** are pointing to cPax6 expressing cells, that appear to migrate tangentially from an extratelencephalic source (the prethalamic eminence, EMT) to populate some parts of the EAce, as it happens in chicken. This stream is also present in mice, but it primarily produces cells for some divisions of the medial extended amygdala (EAme). **h**–**i** High-magnification digital images of frontal telencephalic sections of zebra finch at PHD11 hybridized for cPax6 (**h**), and for cpENK (**i**). Note that cPax6 expression is already weak at PHD11 (compare cPax6 in panels H and D), while cpENK expression is stronger compared to prehatching stages (Fig. 2). For abbreviations, see list. *Scale bars* *A* = 500 µm (applies to **a**–**c**); *D* = 500 µm (applies to **d**–**g**); *H* = 300 µm (applies to **h**–**i**)

**Fig. 4:**
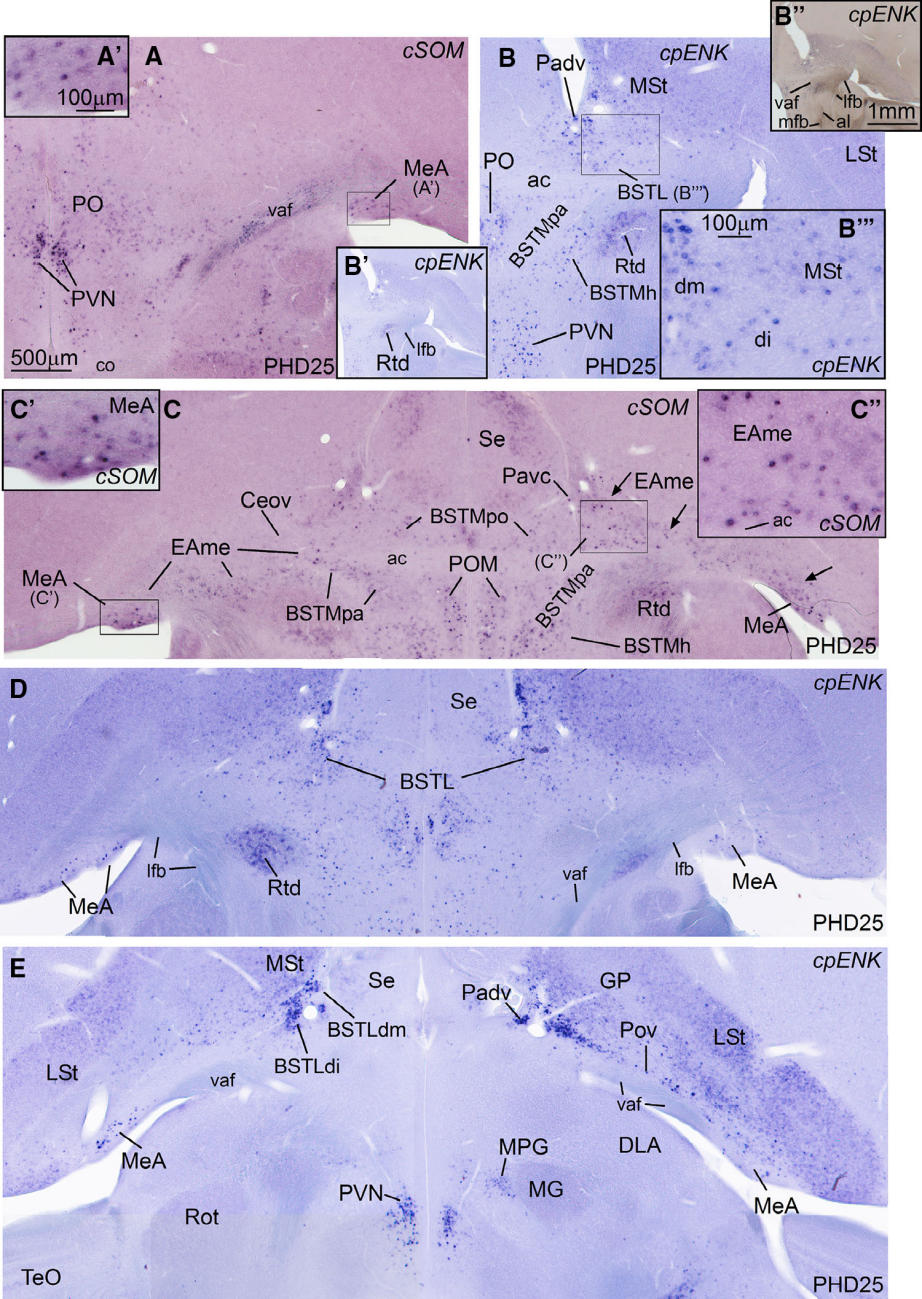
Expression of cSOM and cpENK in the the central and medial extended amygdala of the zebra finch juveniles (PHD25). **a**–**e** High-magnification digital images of horizontal telencephalic sections of zebra finch at PHD25 hybridized for cSOM (**a** and **c**), or for cpENK (**b**; **d**–**e**). Panoramic views of the section of panel **b** are shown in **b′** and **b″**: these are two images of the same section, but taken with different light intensity, so that the signal and other aspects of the tissue differ slightly; for example, the fiber tracts are noticed in **b″**, facilitating a better comprehension of the topological location of the cell groups expressing cpENK. **a′**, **b′′′**, **c′** and **c″** show details of cSOM (**a′**, **c′** and **c″**) and cpENK (**b′′′**)-expressing cells in the MeA (**a′** and **c′**), BSTLd (**b′′′**, medial and intermediate parts of BSTLd are labeled as dm and di, respectively) and periventricular parts of EAme (**c″**; which include part of the BSTM). The *arrows* in **c** points to a cSOM-expressing cell corridor of the EAme, extending from periventricular levels of the ventrocaudal pallidal domain (where a dorsal part of BSTM locates) to the MeA (laterally). A ventral branch of this cell corridor extends into the ventral aspects of BSTM. **d** shows a section at the level of BSTLd and POM, while E is showing a more caudal section, where Pov and MeA are seen on the right side, while some parts of BSTLd are still present on the left side. Note the cell corridor of cpENK cells extending from the dorsoventral pallial domain lateralwards throughout the Pov; this cell corridor runs parallel and dorsally to that of the SOM cells of the EAme (compare **e** with **c**). For abbreviations, see list. *Scale bars* *A* = 500 µm (applies to **a**–**e**); *A′*= 100 µm; *B′′* = 1 mm (applies to **b′** and **b″**); *B′″* = 100 µm (applies to **b′′′**, **c′** and **c″**)

**Fig. 5:**
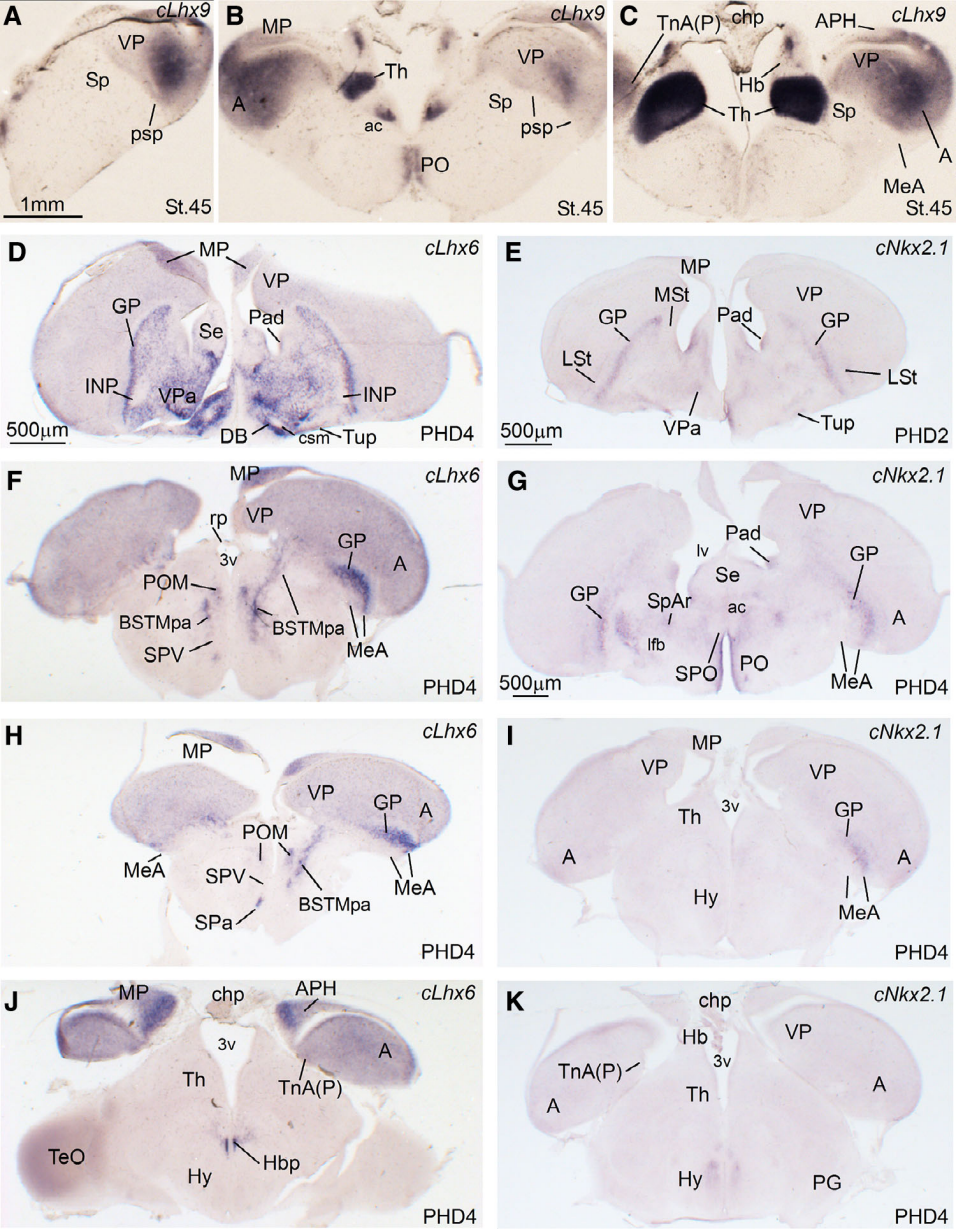
Expression of cLhx9, cLhx6 and cNKx2.1 in the telencephalon of zebra finch embryos at pre-hatching stages (St. 45), and post-hatching days 2 and 4 (PHD2, PHD4). **a**–**k** Low-magnification digital images of frontal telencephalic sections of a zebra finch embryo hybridized for cLhx9 (**a**–**c**), and oblique-horizontal telencephalic sections of a PHD4 zebra finch hybridized for cLhx6 (**d**, **f**, **h** and **J**), and PHD2 (**e**) and PHD4 (**g**, **i**, and **k**) zebra finches hybridized for cNkx2.1. cLhx9 is expressed by medial (MP) and ventral (VP) pallial derivatives, including the arcopallium (**a**). Nucleus taeniae (TnA) is a pallial nucleus that develops within the arcopallial complex, being rich in cLhx9, but poor in expression of subpallial genes (as cLhx6) (as a reference, the pallio-subpallial boundary, psp, is indicated in **a** and **b**). In the subpallium, we identified the medial amygdala (MeA) of zebra finch, having a similar location and genetic profile to that of chickens and mice. It contains a pallidal subdivision rich in cNkx2.1 and cLhx6 (**f**–**i**), but poor in cLhx9 (**c**). Note that cLhx6 and Nkx2.1 are expressed in the complete radial pallidal domain (**f**–**h**), having at the surface the pallidal part of MeA (shown in **f**–**i**). When compared with zLhx5 expression (see Fig. 6), the pallidal component of the MeA seems to be located laterally in the nucleus. Thanks to the oblique (quasi-horizontal) plane employed at posthatching, it is possible to see a stream or cell corridor of cLhx6-expressing cells extending ventrally from the periventricular region of the ventrocaudal pallidal domain, through the pallidal part of the BSTM (BSTMpa) (shown in **f** and **h**). For abbreviations, see list. *Scale bars* *A* = 1 mm (applies to **a**–**c**); *D* = 500 µm; *E* = 500 µm (applies to **e** and **f**); *G* = 500 µm (applies to **g**–**k**)

**Fig. 6:**
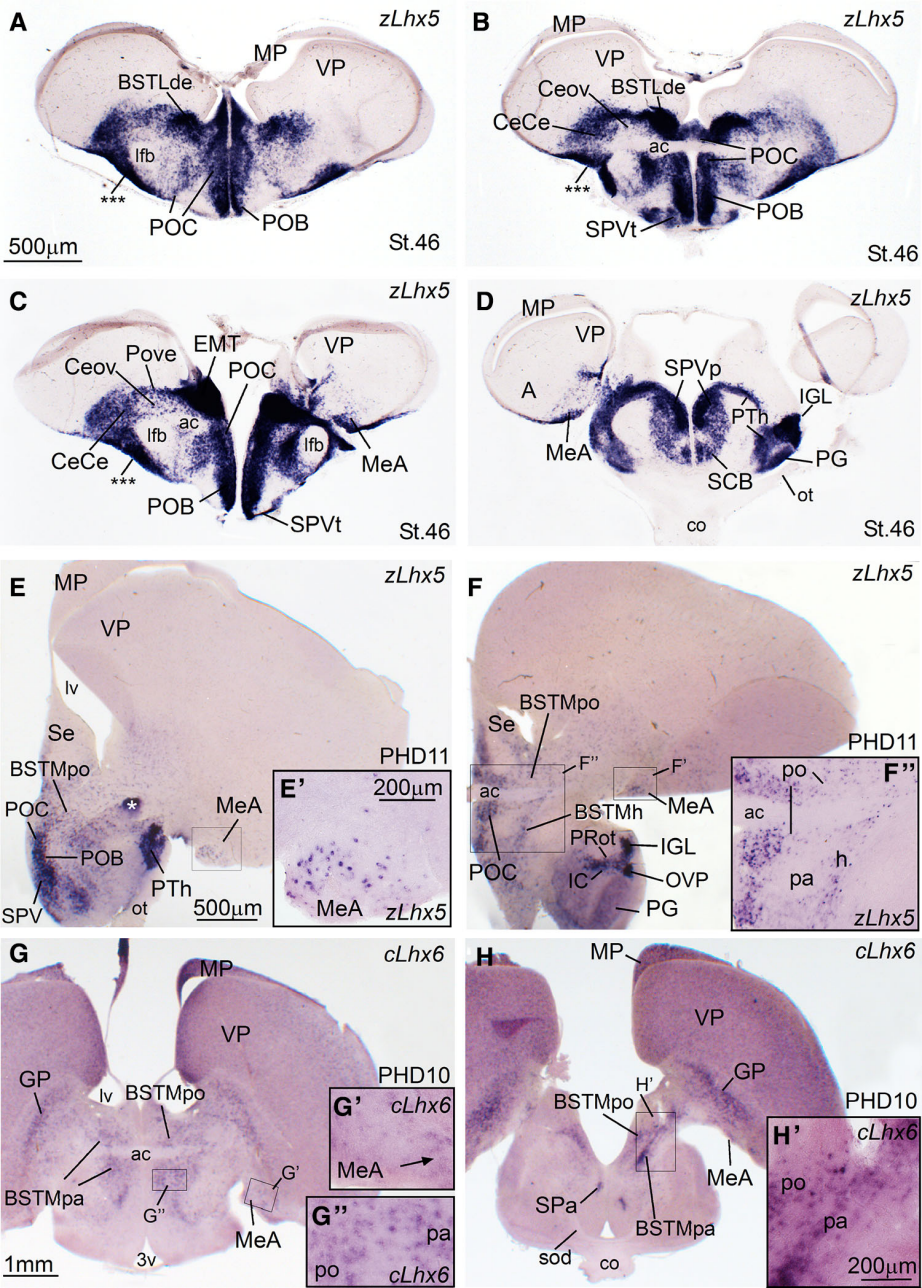
Expression of zLhx5 and cLhx6 in the telencephalon of zebra finch embryos at pre-hatching stages (St. 46), and post-hatching days 10 and 11 (PHD10, PHD11). **a**–**h** Low-magnification digital images of frontal telencephalic sections of zebra finch embryo (**a**–**d**) and oblique-horizontal telencephalic sections of juveniles (**e**–**f**) hybridized for zLhx5 (PHD11, panels **e**, **f**; details in **e′** and **f″**) or cLhx6 (PHD10, panels **g**, **h**; details in **g′**, **g″**, **h′**). Sections from intermediate (**a**) to caudal (**d**) levels are shown in **a**–**d**. cLhx5 is strongly expressed in the prethalamic eminence (EMT), and in large subpopulations of cells that appear to migrate tangentially to the telencephalon, invading different parts of the central and medial extended amygdala (also the olfactory tubercle, as indicated by the *asterisks* in panel **a**). The extratelencephalic (EMT) cell components of the different central extended amygdala subdivisions are labeled with the suffix “e”, as follows: of CeCe (**b** and **c**), Pove (**c**), BSTLde (**a**, **b**). The medial extended amygdala (EAme), including MeA (**c**, **e** and **f**) and BSTM (**e**, **f**) also include large subpopulations of cLhx5 expressing cells. However, in the case of EAme, these cells may partially come from other domains, such as the preoptic region (PO) and the SPV hypothalamic domain. Note the organization of the BSTM in parallel cell corridors or stripes of different genetic profile and possibly origin: a medial, preoptic corridor (BSTMpo; expressing zLhx5 and cLhx6; **e**–**g**); an intermediate, pallidal corridor (BSTMpa; expressing cLhx6, but not zLhx5; **f**–**h**; see details in **f″** and **h′**); and a lateral hypothalamic corridor (BSTMh, expressing Lhx5, but not Lhx6; **f**, **f″**). As noted above, part of the zLhx5 cells of BSTM may come from EMT, but the location of such cells with respect to the BSTMh corridor is unclear. G and H show the cLhx6 expressing pallidal component of MeA at PHD10 (**g**; detail in **g′**; cLhx6 expressing cells are pointed with an *arrow*). For abbreviations, see list. *Scale bars* A = 500 µm (applies to **a**–**d**); *E* = 500 µm (applies to **e** and **f**); *G* = 1 mm (applies to **g** and **h**); *E′* = 200 µm (applies to **e′**, **f″**, **g′** and **g″**); *H′* = 200 µm

**Fig. 7:**
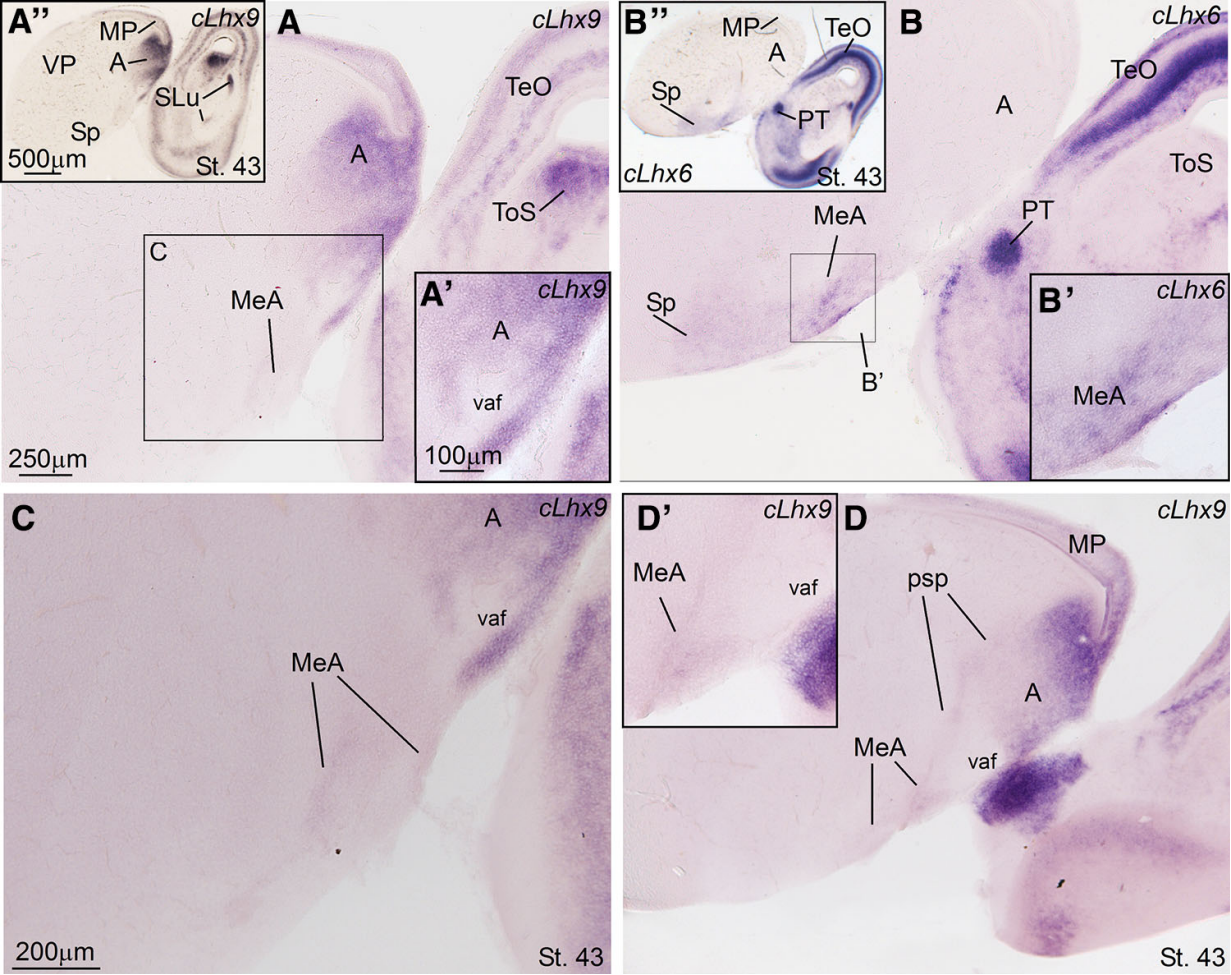
Comparison of cLhx9 and cLhx6 expression in sagittal sections of the telencephalon of zebra finch embryos (St. 43). **a**–**d** High-magnification digital images of sagital telencephalic sections of a zebra finch embryo hybridized for cLhx9 (**a**, **c**–**d**) or cLhx6 (**b**). Panoramic views of the sections are shown in **a″** for cLhx9 and **b″** for cLhx6, while details of the medial amygdala (MeA) are shown in **c** for cLhx9, and **b′** for cLhx6. **a′** is a detail of the rostral pole of the arcopallium (**a**), where the ventral amygdalofugal tract (vaf) is apparent. **c** Is a more ventral detail, including the arcopallium and the MeA (in the subpallium), which is poor in cLhx9. **d** Is a medial section, where the pallio-subpallial border (psp) is seen, and **d′** is showing a detail of the MeA, where cLhx9 is expressed in a very subdued manner, possibly in relation to a very minor subpopulation of immigrant cells coming from the pallium, similarly to that described in mice, chickens, and lizards. For abbreviations, see list. *Scale bars* *A* = 250 µm (applies to **a**, **b** and **d**); *A′* = 100 µm (applies to **a′**, **b′** and **d′**); *A′′* = 500 µm (applies to **a″** and **b″**); *C* = 200 µm

**Fig. 8:**
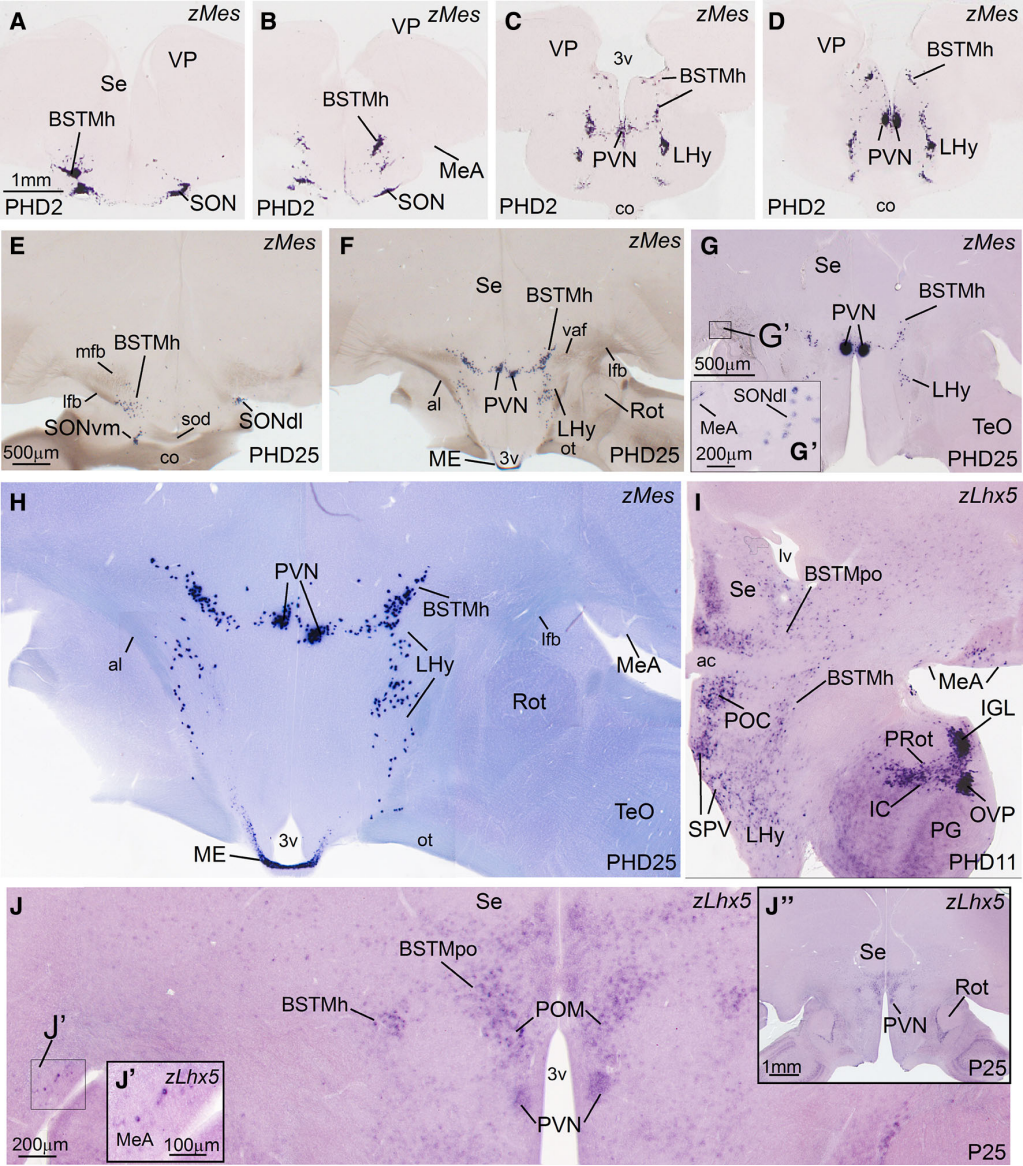
Expression of zMes and zLhx5 in the telencephalon of zebra finch at post-hatching days 2, 11 and 25 (PHD2, PHD11 and PHD25). **a**–**g** Low-magnification digital images of oblique (**a**–**d**) or horizontal (**e**–**j**) telencephalic sections of zebra finch at PHD2 (**a**–**d**) and PHD25 (**e**–**g**) hybridized for zMes. H is showing a detail of the section shown in **f**, focussed on the zMes cells of the BSTM. **i** and **j** are high-magnification images of frontal sections of zebra finch at PHD11 (**i**) and PHD25 (**j**), hybridized for cLhx5, at the level of BSTM and MeA. **j″** is a panoramic view of the section shown in **j**, whereas **j′** is a detail of the MeA. Comparison of zMes (**f**–**h**) and zLhx5 (**i**, **j**) suggests that the mesotocin cells of the BSTMh originate in the SPV hypothalamic domain, the same domain that produces the mesotocin cells of the paraventricular hypothalamic nucleus (PVN) and lateral hypothalamus (Lhy). For abbreviations, see list. *Scale bars* *A* = 1 mm (applies to **a**–**d**); *E* = 500 µm (applies to **e** and **f**); *G* = 500 µm (applies to **g**–**i**); *G′* = 200 µm; *J* = 200 µm; *J′* = 100 µm; *J′′* = 1 mm

**Fig. 9:**
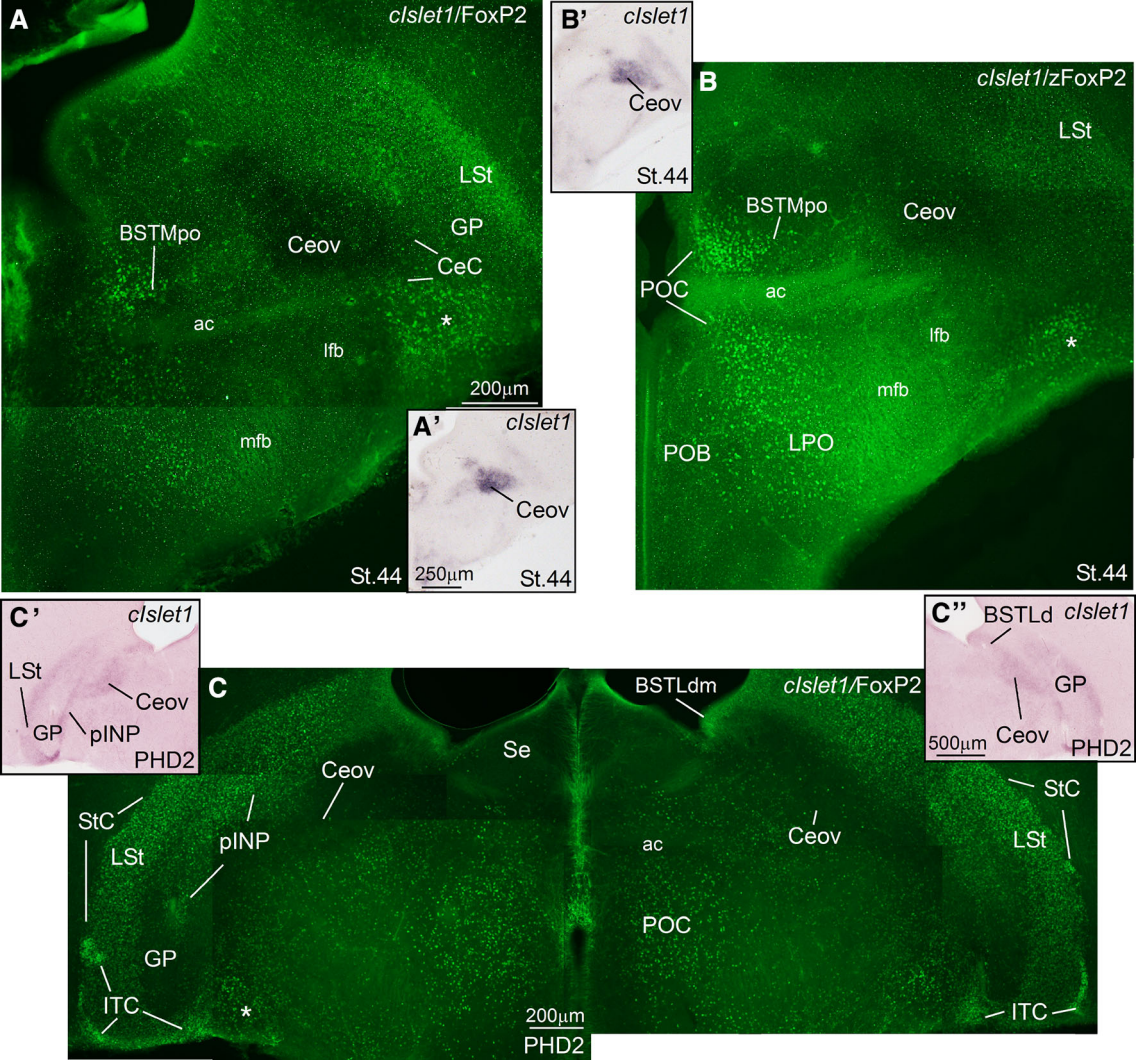
Double-labeling of FoxP2 and cIslet1 in the extended amygdala of a zebra finch at embryonic stage 44 (St. 44) and at post-hatching day 2 (PHD2). **a**–**c** High-magnification digital images of frontal telencephalic sections of zebra finch at St44 (**a**–**b**) and at PHD2 (**c**) hybridized for cIslet1 and immunolabeled for FoxP2. In these double-labeled sections (taken at the level of the central extended amygdala), the hybridization signal is seen in dark on the tissue when using the fluorescence microscope (panels **a**, **b**, **c**; note the dark signal in Ceov; the FoxP2 fluorescence is seen in *green*). To clarify the location of the hybridization signal (for the mRNA-expression pattern), the sections were photographed using bright field microscopy, and panoramic digital pictures are shown in the small panels adjacent to each immunofluorescence image (**a′** for **a**; **b′** for **b**; **c′** and **c′′** for **c**). FoxP2 is present in several subdivisions of the central extended amygdala, such as the StC, the ventral ITC-like patches, the pINP, the CeC and the Ceov. Among these, FoxP2 expression was particularly abundant in intercalated cell groups (StC and ventral ITC), but only few FoxP2 expressing cells were seen in CeC and Ceov. In the medial extended amygdala, FoxP2 was abundant in the preoptic part of BSTM (BSTMpo) (**a** and **b**). The *asterisk* in **a**, **b** and **c** is showing a stream of cells showing strong expression of FoxP2, which appear to come from the prethalamic eminence (EMT). These cells appear to correspond, at least in part, to the cPax6-expressing cells (derived from EMT) described in Figs. 2 and 3. Due to their position in relation to other EAme subdivisions, such EMT-derived cells may belong to this system. For abbreviations, see list. *Scale bars* *A* = 200 µm (applies to **a**–**b**); *A′* = 250 µm (applies to **a′**–**b′**) *C* = 200 µm; *C′′* = 500 µm (applies to **c′** and **c′′**)

**Fig. 10:**
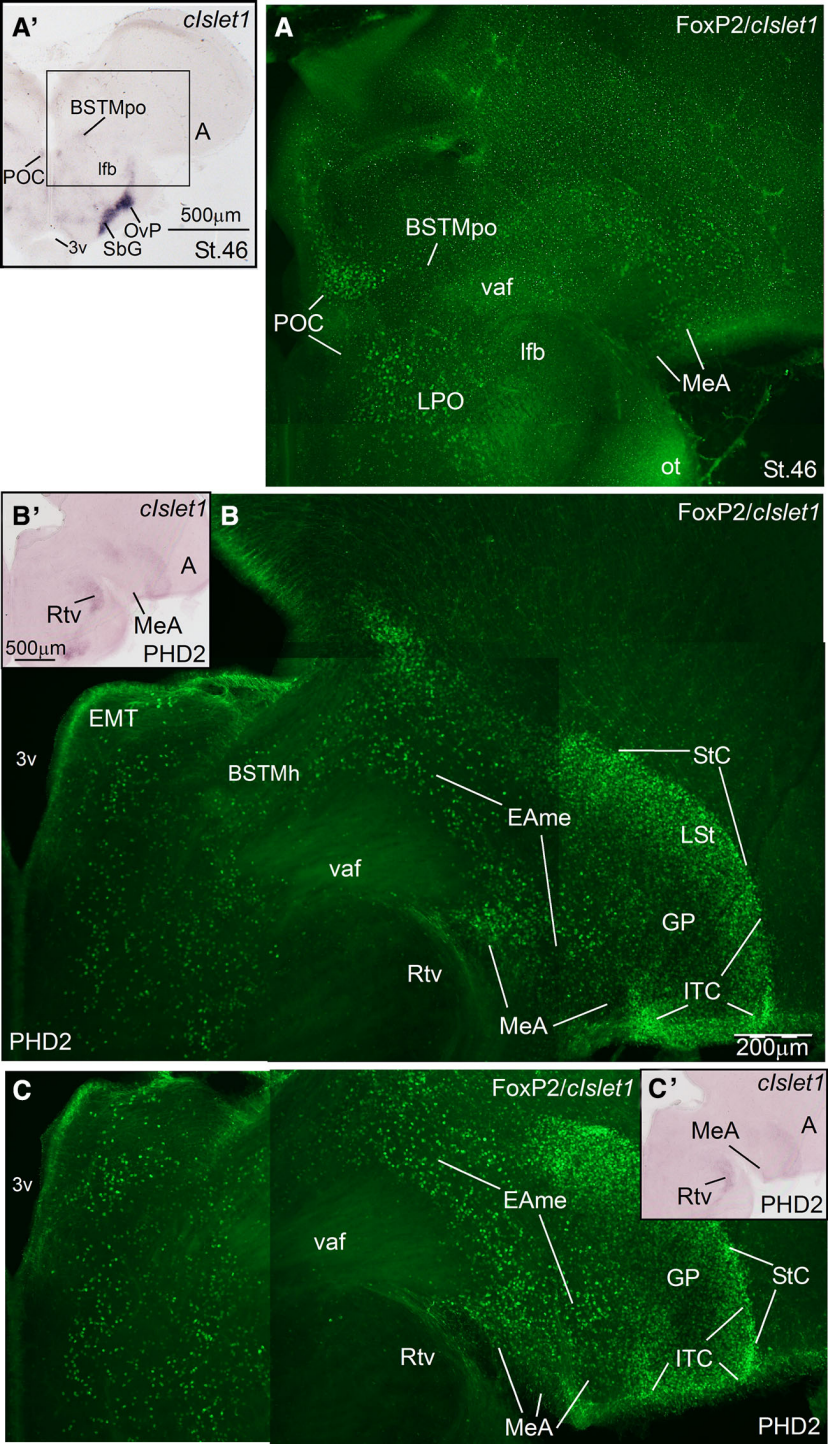
Double-labeling of FoxP2 and cIslet1 in the extended amygdala of a zebra finch prehatch embryo (St. 46) and a post-hatchling at PHD2. **a**–**c** High-magnification digital images of oblique telencephalic sections of zebra finch at embryic St46 (**a**) and at PHD2 (**b** and **c**), hybridized for cIslet1 (seen in *dark*) and immunolabeled for FoxP2 (seen in *green*). For better visualization of the hybridization signal, panoramic digital pictures of the sections using brightfield microscopy are shown in panels **a′**–**c′** (**a′** for **a**; **b′** for **b** and **c′** for **c**). Strong FoxP2 expression is seen in the intercalated cells (StC and ventral ITC). In addition, numerous FoxP2-expressing cells are present in the several subdivisions of the medial extended amygdala, including the preoptic BSTM (BSTMpo) (**a**), the hypothalamic BSTM (BSTMh) (**b**, **c**), and the MeA (**a**, **b** and **c**). Note the expression of FoxP2 in many cells of the prethalamic eminence (EMT). For abbreviations, see list. *Scale bars* *B* = 200 µm; (applies to **a**–**c**); *A′* = 500 µm; *B′* = 500 µm (applies to **b′**–**c′**)

**Fig. 11:**
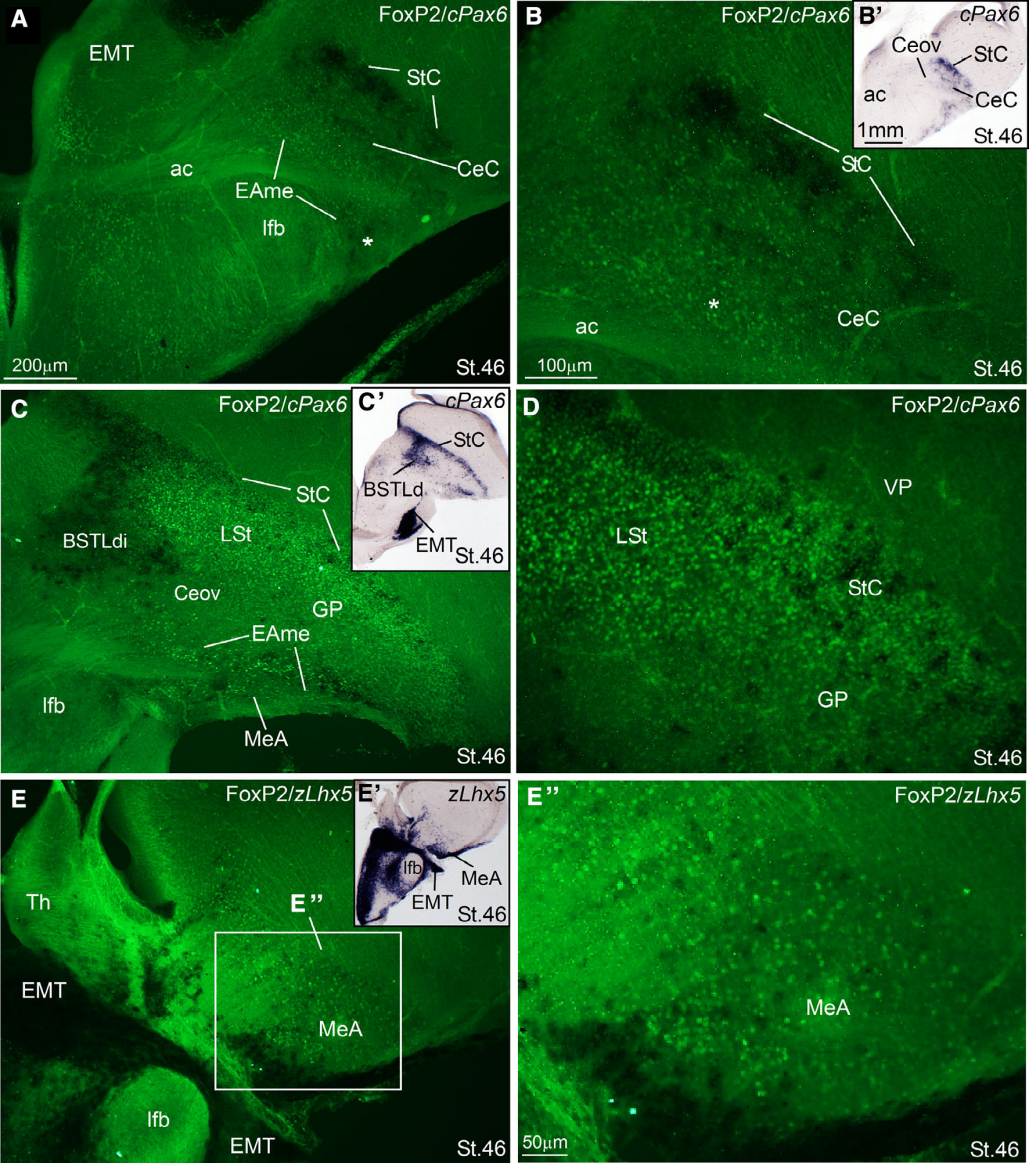
Double-labeling of FoxP2 and either cPax6 or zLhx5 in the extended amygdala of zebra finch embryos at a prehatching stage (St. 46). **a**–**e** High-magnification digital images of oblique (quasi-horizontal) telencephalic sections of zebra finch embryos, hybridized for cPax6 and immunolabeled for FoxP2 (**a**–**d**), or hybridized for zLhx5 and immunolabeled for FoxP2 (**e**). The hybridization signal is seen in *dark*, while the immunofluorescence is seen in *green*. For better visualization of the hybridization signal, panoramic digital pictures of the sections, taken using bright-field microscopy, are shown in panels **b′**, **c′** and **e′** (**b′** for **a** and **b**; **c′** for **c** and **d**; **e′** for **e**). Cells expressing FoxP2 overlap with the cPax6-expresing cells of the intercalated areas (like StC) (**c**, **d**) and the CeC (**a**, **b**), which appear to primarily derive from the dorsal striatal division. This also happens in parts of the medial extended amygdala (EAme), where the Pax6 cells may primarily (if not exclusively) derive from prethalamic eminence (EMT) (note the ovelap in the area labeled with an *asterisk* in **a** and **b**; and in the MeA in panels **c**, **e′**, **e′′**). For abbreviations, see list. *Scale bars* *A* = 200 µm; (applies to **a**, **c** and **e**); *B* = 100 µm (applies to **b** and **d**); *B′* = 1 mm (applies to **b′**, **c′** and **e′**); *E′′* = 50 µm

**Fig. 12:**
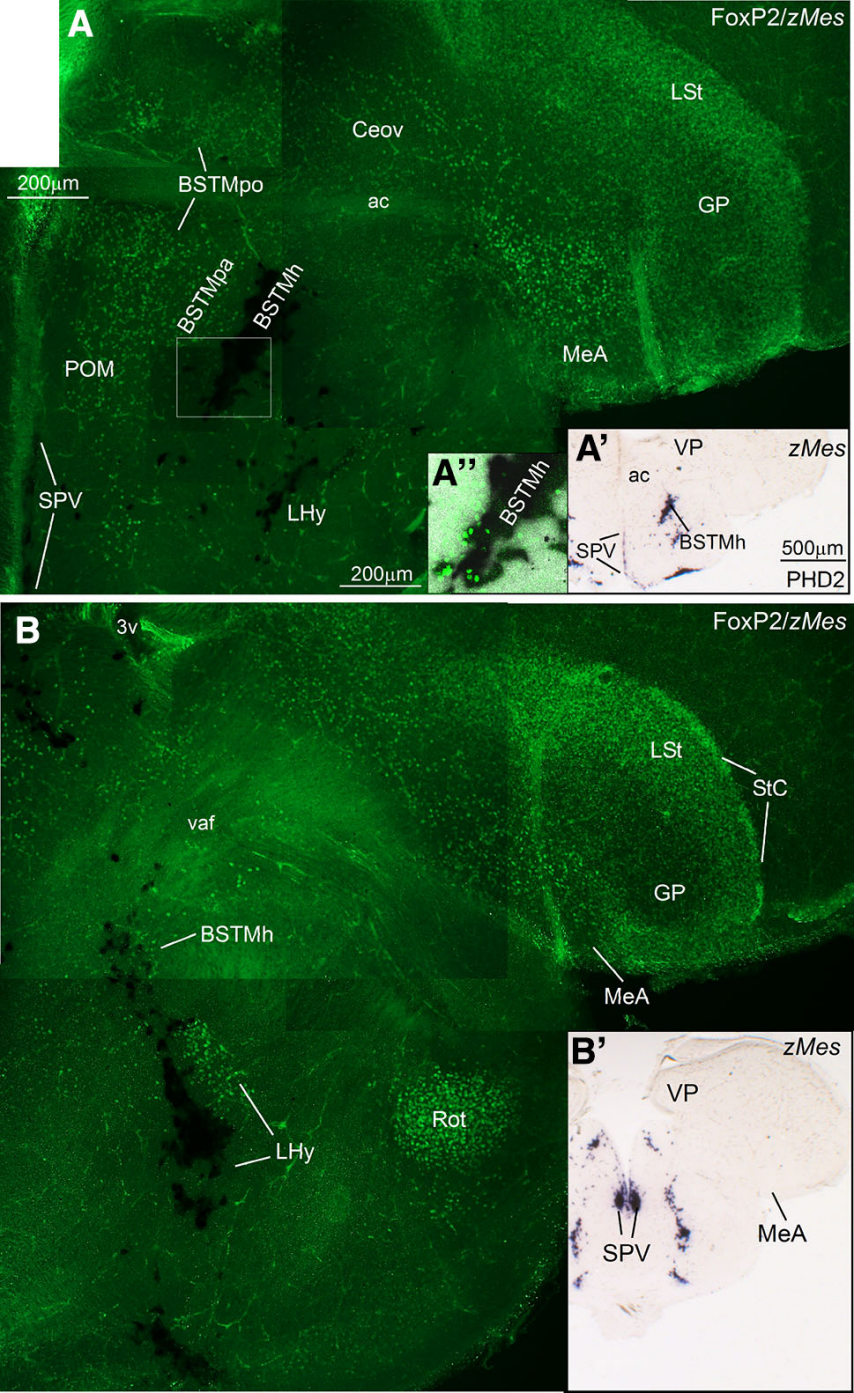
Double-labeling of FoxP2 and zMes in the extended amygdala of a zebra finch post-hatchling (at PHD2). **a**–**b** High-magnification digital images of oblique (quasi-horizontal) telencephalic sections of a zebra finch posthatchling, hybridized for zLhx5 (*dark*) and immunolabelled for FoxP2 (*green*). For better visualization of the hybridization signal, panoramic digital pictures of the sections, taken with brigh field microscopy, are shown in panels **a′** and **b′** (**a′** for **a** and **b′** for **b**). Note the presence of FoxP2 expressing cells in all parts of the BSTM, including the preoptic (BSTMpo), the pallidal (BSTMpa) and the hypothalamic (BSTMh) subdivisions. In the latter, FoxP2 cells overlap with those expressing mesotocin (detail in **a′′**; also panel **b**). Overlap of FoxP2 cells and zMes cells also occurs in the hypothalamus. Analysis at high magnification with the confocal microscope suggest co-expression of mesotocin and FoxP2 in cells of the lateral hypothalamus (not shown). Note also the expression of FoxP2 in other parts of the extended amygdala (StC, MeA), in the lateral striatum (LSt) and in the thalamic nucleus rotundus (Rot). For abbreviations, see list. *Scale bars* *A* = 200 µm; (applies to **a** and **b**); *A′* = 500 µm (applies to **a′** and **b′**)

**Fig. 13:**
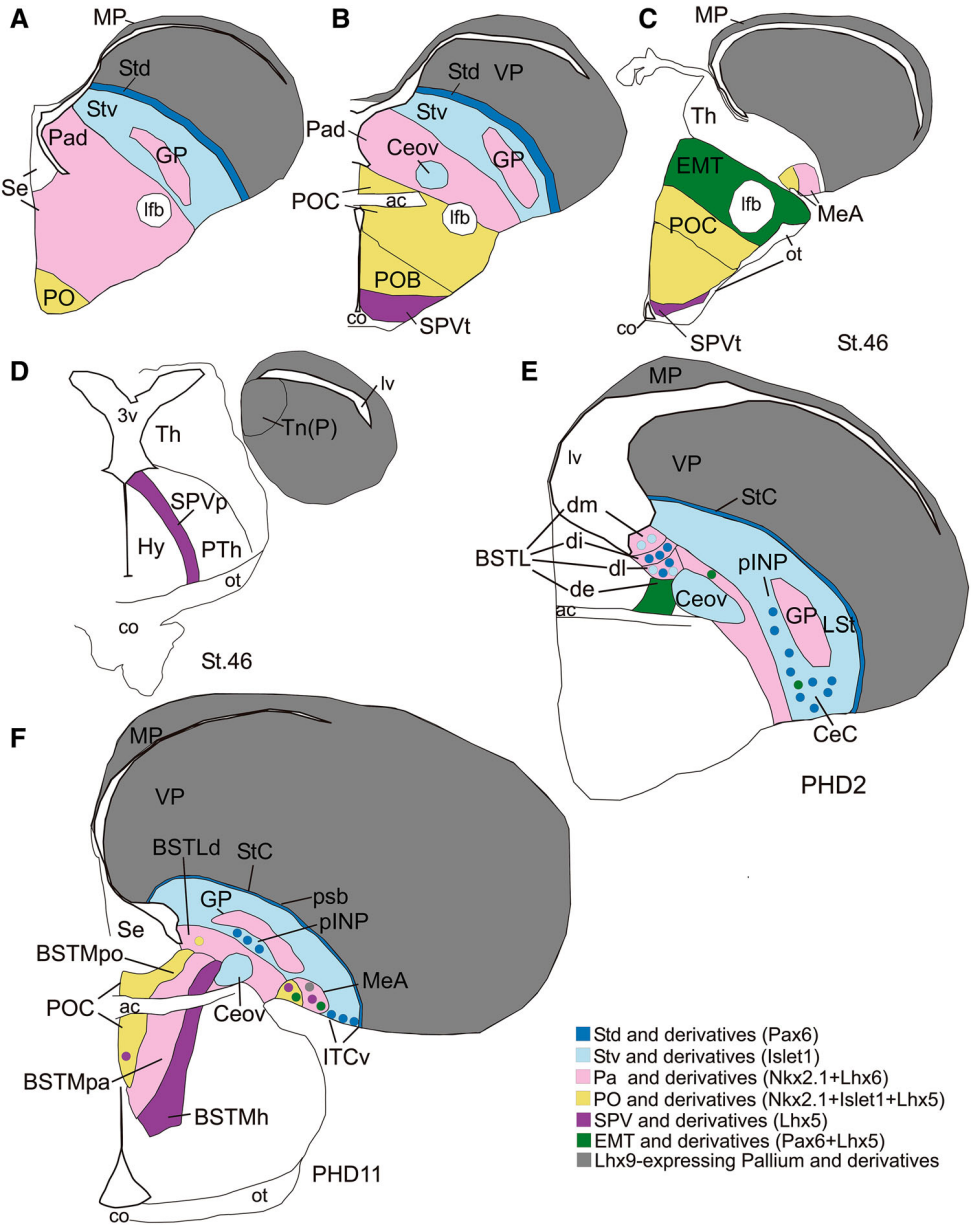
Schematic drawings of finch brain sections representing the main subdivisions of the central and medial extended amygdala and their embryonic origin. **a**–**d** are schematics of frontal forebrain sections of St.46 embryos from rostral (**a**) to caudal (**d**) levels. The whole pallium is shown in *grey*, while different progenitor domains of the subpallium and outside the telencephalon that contribute cells to the extended amygdala are shown in *different colors*. **e** and **f** are schematics of frontal sections of the posthatch brain at the level of the central extended amygdala (**e**) or the medial extended amygdala (**f**), representing some of the major subdivisions with respect to the radial histogenetic domains, as well as immigrant cell subpopulations with different origin (*labeled with different colors*). For abbreviations see list

Next, we present frontal (in embryos) and quasi-horizontal (posthatchlings, juveniles) brain sections at the level of the central extended amygdala (EAce), hybridized for cIslet1, cPax6, cNkx2.1, cpENK, and cSOM (Figs. 2, 3, 4), and sections with the same planes at the level of the medial extended amygdala (EAme), hybridized for cNkx2.1, cLhx6, cLhx9, zLhx5, and zMes (Figs. 5, 6, 7, 8). cSOM and cpENK shown in Fig. 4 were also useful for distinguishing some cell subpopulations of EAme. In Figs. 9, 10, 11, 12 we present data on the expression of the transcription factor FoxP2 (using immunofluorescence to label the protein; seen in green), done for comparison purposes on sections hybridized for Islet1, Pax6, Lhx5, and Mes (dark signal; the hybridization signal is seen in bright field in the insets adjacent to each fluorescence microscopy image). The last figure (Fig. 13) presents schematics of finch brain sections at the level of the extended amygdala summarizing the main results.

The transcription factors analyzed showed moderate or intense expression during late embryonic stages (St45, St46) and in early post-hatchlings, but declined afterwards. For example, Islet1 expression was stronger in late embryos (Fig. 2a–c) than in post-hatchlings, and was not detected after PHD2 (Fig. 3a–c; at this stage it was rather weak in the subpallium, compared to the prethalamus). This was also the case for cNkx2.1 and cLhx9. However, cPax6, zLhx5 and cLhx6 maintained their expression in the zebra finch subpallium until PHD10/11 approximately. Later, analysis was based on the expression of neuropeptide genes.

Notably, the combinatorial expression of the transcription factors Pax6, Islet1, Nkx2.1, Lhx5, Lhx6, and Lhx9 (Figs. 1, 2, 3, 5, 6, 13), analyzed within the brain topological framework (Nieuwenhuys 1998), allowed the distinction of the same embryonic domains that produce cells for the extended amygdala in mice and chicken, which are the dorsal striatal domain (Std, which produces cells expressing of Pax6), the ventral striatal domain (Stv, which produces cells expressing Islet1), the pallidal embryonic domain (Pa, which produces cells expressing Nkx2.1 and Lhx6), the preoptic embryonic domain (PO, which produces cells expressing Nkx2.1, Islet1, and Lhx5), the ventrolateral caudal pallium (which produces cells expressing Lhx9), the supraopto-paraventricular hypothalamic domain (SPV, which produces cells expressing Lhx5), and the prethalamic eminence (EMT, which produces cells expressing Pax6 and Lhx5) (Fig. 13). Some of these domains could be further subdivided based on the expression of genes encoding different neuropeptides, such as proenkephalin (pENK), somatostatin (SOM or SST), and mesotocin (Mes), as explained below. Most of the above-mentioned embryonic divisions of zebra finch were similar to those of the chicken, although variations in their relative size were observed. For example, the zebra finch dorsal pallidal embryonic domain (Pad) was very prominent, much more than in chicken, producing a sort of ventricular eminence (Fig. 2g–i) that—interestingly—resembled the medial ganglionic eminence of mammals.

## Central extended amygdala (EAce) of the zebra finch

For determining the location and extension of the different areas of the EAce in zebra finch, we analyzed the combinatorial expression patterns of the transcription factors Pax6, Islet1 and Nkx2.1, which allowed the distinction of dorsal striatal (Std, with Pax6), ventral striatal (Stv, with Islet1), and pallidal (Pa, with Nkx2.1) derivatives. For better identification of specific cell groups, we added FoxP2, pENK and SOM to the analysis, as explained below. Based on the analysis of these genetic markers within the topological framework, the zebra finch EAce is located in the caudal subpallium and can be understood as a structure divided into two major parts: lateral and medial. The lateral component is fundamentally formed by striatal derivatives (from both Std and Stv), and includes the central amygdala and other subdivisions. The medial division is formed within the dorsal pallidal domain and encompasses the dorsal part of the lateral bed nucleus of the stria terminalis (BSTLd). Although these two major parts of EAce contain a majority of either striatal or pallidal cells, they also include subpopulations of immigrant cells, as described below.

Within the lateral part of the finch EAce (late embryos and post-hatchlings), we have identified seven subdivisions comparable to those found in chicken (Vicario et al. 2014):

- Two intercalated (ITC)-like areas, rich in expression of Pax6, pENK, and FoxP2, and apparently derived primarily from the dorsal striatal embryonic domain. These areas are located along the dorsolateral margin of the striatum and capsular central amygdala, and include a dorsal part named striatal capsule (StC) and a ventral, patchy part (Figs. 2d–f, 3d–f for Pax6; Fig. 2k, l for pENK; Fig. 10b, c for FoxP2).
- Three central amygdala-like subdivisions, rich in Pax6 and/or Islet1 cells, apparently derived from the dorsal (Pax6; Std) or ventral (Islet1; Stv) striatal domains. These include the capsular central amygdala (CeC), the central oval nucleus (Ceov) and the peri-intrapeduncular nucleus (pINP). The CeC is located in the caudolateral part of the radial striatal division, and contains Pax6 and pENK expressing cells (Fig. 2f, l). The Ceov is a compact cIslet1-expressing cell mass that is located above the lateral branch of the anterior commisure, medial to CeC, lateral to the dorsal BSTL (BSTLd), and below the pINP and globus pallidus (Figs. 2b, c, 3b, c). Although rich in Islet1 cells of apparent striatal origin, the Ceov appears tangentially displaced ventralwards, being located in the radial pallidal domain. In spite of this location, the Ceov is poor in Nkx2.1 cells of pallido-preoptic origin (Fig. 2i). The finch pINP is located in the radial striatal domain just caudal to the intrapeduncular nucleus, ventral to the globus pallidus and above the Ceov, and it contains many cells expressing Islet1 apparently derived from Stv (Figs. 2b, 3a, b), and many Pax6 cells that mostly appear to derive from Std (Fig. 3f). In addition, the pINP contains a few cells expressing somatostatin (not shown).
- We tentatively identified the subpallial amygdaloid rostral area (SpAr) in Fig. 2b, as an area located in the dorsal pallidal domain (rich in Nkx2.1; Fig. 2g, h), lateral to rostral levels of BSTLd, and also containing some Islet1-expressing cells (Fig. 2b). The SpAr appears located medially to the rostral pole of Ceov.
- The perioval zone (Pov) is a component of the EAce located in the pallidal domain, as a lateral extension of the BSTLd, with a high content of enkephalinergic (pENK) cells (Fig. 4e). The Pov is seen as a cell corridor of pENK cells dorsally adjacent to another corridor of SOM cells related to the medial extended amygdala (Fig. 4c).

Within the medial EAce, the BSTLd is located in the Nkx2.1-rich pallidal domain (Fig. 2h), but seems to include cells derived from the Std (expressing Pax6, Fig. 2e), the Stv (expressing Islet1, Fig. 2c) and the diencephalic prethalamic eminence [EMT; these cells express Pax6 (Fig. 3f) and/or zLhx5 (Fig. 6c)]. As in chicken, the BSTLd of zebra finch includes three subdivisions containing different proportions of immigrant cells from Std and/or Stv: medial (BSTLdm), intermediate (BSTLdi), and lateral (BSTLdl). The Std-derived Pax6 cells accumulate in the intermediate division (BSTLdi, Figs. 2e, 3d–f), while the Stv-derived cIslet1-expressing cells accumulate, in turn, in the medial division (BSTLdm, see in Fig. 3b), forming a compact group of cIslet1-expressing cells adjacent to the ventricular zone of the dorsal pallidal domain. Std- and Stv-derived cells (with Pax6 or Islet1) loosely intermingle in the lateral division (BSTLdl, Fig. 2c).

In addition, in the finch BSTLd we have found another subdivision rich in cells that apparently derive from the EMT (BSTLde). The EMT-derived Pax6 cells in zebra finch can be easily followed into the extended amygdala region (arrow and asterisk in Fig. 3d–f). Some of these Pax6 cells can be followed into the BSTLde, but also into the CeC/ventral ITC-like region, and into the olfactory tubercle (asterisk in Fig. 2d–f). In addition, thanks to the very prominent expression pattern of zLhx5 in the finch, we were able to follow cells from EMT to the BSTLde (Fig. 6a, b), as well as to other components of the EAce, including the Pov, the Ceov, and the CeC. We added the suffix—to label this specific component of these EAce subdivisions: Pove, Ceove and CeCe (Fig. 6b, c).

As other parts of EAce, the BSTLd in zebra finch contains subpopulations of cells expressing proenkephalin (pENK), and such expression was seen from embryonic stages (Fig. 2) until juvenile stages (Fig. 4). Based on the distribution of the cells in zebra finch during development, comparison to region-specific genes (Pax6, Nkx2.1) and comparison to chicken (see “Discussion”), the pENK cells of the BSTLd may have at least three origins: based on comparison with Pax6, part of the pENK cells of BSTLd may originate in Std, as those seen in StC (Fig. 2k); other pENK cells in BSTLd appear to originate in a dorsoventral pallidal subdivision, as those of Pov (Padv; Figs. 3i, 4e); finally, at least a few cells of the caudolateral BSTLd may originate in PO (Fig. 4b).

In addition, a subpopulation of cells expressing somatostatin is seen in parts of the extended amygdala in juvenile zebra finch (PHD25, Fig. 4a, c). Although most of such cells are located in the medial extended amygdala (explained in next section; Fig. 4a, c), a few of them are present in the BSTLd (not shown). These cells may originate in the ventrocaudal pallidal domain (Pavc) (Fig. 4c) (see “Medial extended amygdala (EAme) of the zebra finch” and “Discussion”).

## Medial extended amygdala (EAme) of the zebra finch

For determining the location and extension of the different areas of the EAme in zebra finch, we analyzed the combinatorial expression patterns of the transcription factors Nkx2.1, Lhx6, Lhx5, Lhx9, Islet and Pax6. This allowed the identification of cell subpopulations with different origin, including pallial (Lhx9), pallidal (Nkx2.1 and Lhx6), preoptic (Nkx2.1, Islet1, Lhx5), hypothalamic (from SPV; Lhx5) and prethalamic (from EMT; Pax6, Lhx5).

Our first objective was to identify the medial amygdala of zebra finch. In other vertebrates such as mice and chickens, this is complex nuclear structure located in the caudolateral and ventral aspect of the subpallium, rich in cells of pallidal and preoptic origins, but also containing subpopulations of immigrant cells of ventral pallial (minor), hypothalamic SPV, and EMT origins (reviewed by Medina et al. 2011; Abellán et al. 2013). In zebra finch, we identified a comparable structure in the caudolateral subpallium, which contained a subdivision rich in Lhx6 and Nkx2.1 of apparent pallidal origin, based on its radial alignment with the globus pallidus (MeA, Fig. 5f–i at PHD4; the expression was still present at this age, although decreased at later posthatch stages: Fig. 6g, h). The finch MeA also includes subpopulations of cells expressing Lhx5 (Fig. 6c–f; see details in Fig. 6e′, f′), which may include preoptic, hypothalamic (SPV) and EMT derivatives, as is the case in chickens and mice. At PHD10/11, the Lhx6 and Lhx5 cells of MeA occupy mostly separate positions within MeA (compare Fig. 6e, f with Fig. 6g, h). Later in development, the finch MeA was seen to contain an abundant subpopulation of SOM cells (Fig. 4a, a′, c, c′), which appears to derive from the ventrocaudal pallidal domain (Pavc; cell corridor from Pavc to MeA is labeled with arrows in Fig. 4c), resembling the situation in mice and chickens. The cell corridor of SOM cells spreading from the Pavc to MeA is parallel to ventral amygdalofugal tract (vaf; Fig. 4e) and to another cell corridor of pENK cells (located deeper) spreading from Padv through the Pov (see above; Fig. 4e). The latter cell corridor, originated from Padv, may be the source of at least some of the few pENK cells seen in the finch MeA (Fig. 4d, e).

In addition, our data show that nucleus taeniae (TnA) of zebra finch develops in the caudal ventral pallium, as part of the arcopallial complex (Fig. 5c; note the topological location of this nucleus above the pallio-subpallial border, indicated by the limit of Lhx9 expression). For this reason, here we labeled this nucleus as TnA(P), to refer to its pallial nature (see “Discussion”). During development, the arcopallium is rich in Lhx9 expression (Figs. 5b, c, 7a), but poor in subpallial genes, such as Nkx2.1 and Lhx6 (Figs. 5f–i; 7b). In agreement with its topological location and origin, TnA(P) is rich in Lhx9 expression (Fig. 5c). In contrast, no expression of Nkx2.1 is evident in the TnA(P) at early stages, and the weak expression of Lhx6 seen at PHD4 in this nucleus likely relates to interneurons (seen throughout the pallium; see “Discussion”). In contrast to TnA(P), the MeA identified here is a subpallial struture (develops below the pallio-subpallial boundary; Fig. 5c), and contains many cells expressing Nkx2.1 and Lhx6 (Figs. 5f–i, 7b, b′), but is poor in Lhx9 (Fig. 5c), except for the presence of a few cells (Fig. 7a, c), which likely emigrate tangentially from the pallium, as described in chickens and mice (see “Discussion”).

Regarding the medial bed nucleus of the stria terminalis (BSTM), based on the expression of different genes we identified in this nucleus several parallel cell corridors or stripes with different genetic profile and perhaps different embryonic origin. Based on Lhx6, we observed a cell corridor of pallidal cells (BSTMpa; Figs. 5f, h, 6g, h) comparable to that described in the BSTM of chickens (Medina and Abellán 2009) and mice (García-López et al. 2008; Bupesh et al. 2011b). This cell corridor, extending ventralwards to almost reach the alar hypothalamus, also contained SOM cells, suggesting its origin in the ventrocaudal pallidal domain (Fig. 4c). This cell corridor is continuous with the cells of the medial extended amygdala that spread into the MeA (Fig. 4c).

The cell corridor of pallidal cells of the BSTM was delineated medially and laterally by other corridors of Lhx5-expressing cells (Fig. 6e, f; detail in f′′). Based on their position and expression of other markers (especially Nkx2.1 and Islet1; Fig. 2i; for Islet1 see Fig. 10a′), the medial cell corridor is likely preoptic (accordingly named BSTMpo). The preoptic cell corridor is dorsally continuous, above the anterior commissure, with cells adjacent to the ventral tip of the lateral ventricle (Fig. 6f, f″). In addition to Lhx5, Nkx2.1, and Islet1 cells, it contains subpopulations of cells expressing pENK (Fig. 4b), SOM (Fig. 4c), and Lhx6 (Fig. 6g). On the other hand, the cell corridor lateral to the BSTMpa appeared to include cells of extratelencephalic origin, part of which seem to derive from the SPV domain of the alar hypothalamus, which also produces the paraventricular hypothalamic nucleus (this subdivision was accordingly named BSTMh). The BSTMh was poor in Lhx6 (Fig. 5f) and SOM cells (Fig. 4c), but contained cells expressing Lhx5 (Figs. 6f, 8i) and pENK (Fig. 4b). Notably, the BSTMh also includes a subpopulation of mesotocin (zMes)-expressing cells, which are continuous with those present in the paraventricular hypothalamic nucleus (PVN) and other parts of the alar hypothalamus (Fig. 8). Based on this, it appears that SPV, which produces the mesotocinergic cells of PVN and supraoptic nucleus (SON), is also the source of those cells that populate the BSTMh (in a more dorsal location). This has also been suggested in mice and chickens (see “Discussion”). Other cells appear to spread ventrally from the SPV domain, to reach ventral parts of the lateral hypothalamus (Fig. 8). Mesotocin expression was observed in all of these groups from early stages (at least PHD2, but it is possibly expressed in SPV derivatives from earlier embryonic stages) and was maintained in juveniles (Fig. 8e–h) and later (unpublished results). Our data also showed that mesotocinergic cells do not appear to reach the MeA in a significantly number, although a few cells appear to be present (see a detail of this area in 8G).

In addition, the BSTM likely includes a subpopulation of Pax6 and Lhx5 cells coming from the EMT, as described in chickens and mice (see “Discussion”). However, our data did not allow determining whether these cells are segregated or overlap with those of other origins (in particular, with those of the hypothalamus).

## FoxP2 expression in different components of the zebra finch’s extended amygdala

As a first approach to understanding the location of FoxP2 in the extended amygdala, we mapped the FoxP2 expression in the zebra finch at early posthatch stages of development (Figs. 9, 10, 11, 12), and compared its expression pattern with those of cIslet1 (Figs. 9, 10), cPax6 (Fig. 11a–d), zLhx5 (Fig. 11e, e′′), and zMes (Fig. 12).

Comparison with Islet1 and Pax6 indicated that FoxP2 is expressed in several areas within the finch EAce (Figs. 9, 10, 11). In particular, FoxP2 was enriched in the ITC-like cells, including its dorsal extension (StC) and the ventral ITC-like cell patches (Figs. 9c, 10b, c, 11c, d). In StC, FoxP2 is expressed in the same areas as Pax6 (shown in Fig. 11c; detail in Fig. 11d), suggesting a common origin of these cells in the Std. Other parts of EAce also contain some FoxP2 expressing cells, such as the CeC (9A), pINP, and Ceov (Fig. 9a–c). These are abundant in pINP (Fig. 9c), resembling the situation in the striatum. However, in the CeC and Ceov, just a few of the cells are FoxP2-positive (Figs. 9a, 11a–c).

Different areas of the zebra finch EAme are also populated by FoxP2-expressing cells, including specific subdivisions of MeA and BSTM. Based on the location of the FoxP2 expressing cells in the forebrain, it appears that these cells concentrate in preoptic, hypothalamic and EMT derivatives, including those in the BSTM and MeA (Fig. 10a–c). Regarding the BSTM, FoxP2 cells were seen in the BSTMpo subdivision (Figs. 9a, b, 10a, 12a), which can be defined because of its location and expression of cIslet1 (Fig. 10a′). In addition to BSTMpo, FoxP2 cells were present in the BSTMh subdivision (10B), extending dorsally in a cell corridor through the EAme reaching the MeA (Fig. 10b, c). In the BSTMh, FoxP2 expressing cells overlapped those containing mesotocin (Fig. 12b); at high magnification, we could observe that both markers colocalized in some cells of the hypothalamus, although we could not see whether this also happens in BSTMh (not shown). Moreover, FoxP2 was present in numerous EMT-derived cells that spread throughout the EAme (asterisk in Figs. 9a–c, 11a, b), also reaching the MeA, where they overlap with cells expressing Pax6 (Fig. 11c) and Lhx5 (Fig. 11e, e″). Finally, FoxP2 was also expressed in cells of the BSTMpa, although such cells were less abundant in this subdivision (Fig. 12a) compared to those in BSTMpo, BSTMh, and the EAme areas enriched in EMT-derived cells.

## Discussion

In this study we used a battery of developmental regulatory genes (encoding region-specific transcription factors) and neuropeptide genes to study the extended amygdala in zebra finches. Zebra finches are a highly gregarious species of songbirds, that learn and use song for social communication (Riters et al. 2004; Fisher and Scharff 2009; Goodson 2013; Wohlgemuth et al. 2014), and are widely employed for social behavior studies (Goodson et al. 2009; Fischer and Hammerschmidt 2011; Kelly et al. 2011; Goodson 2013; Kelly and Goodson 2013, 2014; Kingsbury and Goodson 2014; Boogert et al. 2014; McCowan and Griffith 2015). The extended amygdala is highly relevant for controlling or modulating this behavior (reviewed by Martínez-García et al. 2007), and in many of the studies on the neural basis of social behavior in zebra finches there is specific mention of the medial amygdala (suggested to be the so-called nucleus taeniae) and the BSTM (for example, Goodson et al. 2012; Kelly and Goodson 2013). However, studies in mice and chickens using gene expression data and fate mapping have shown that the extended amygdala includes multiple subdivisions and cell corridors or stripes, each defined by a specific genetic profile and embryonic origin (mouse: García-López et al. 2008; Bupesh et al. 2011a, b; chicken: Abellán and Medina 2009; Vicario et al. 2014, 2015; reviews by Medina et al. 2011; Kuenzel et al. 2011; Abellán et al. 2013). Importantly, each different cell corridor may be engaged in a different functional pathway, and for this reason such developmental studies provide a powerful tool for starting to disentagle amygdalar functional organization, and may help to establish a new paradigm for interpreting functional data and for understanding the neural basis of social behavior (Medina and Abellán 2012; Abellán et al. 2013). However, detailed data on gene expression patterns focused on the amygdala during development were missing in songbirds.

Our data have helped to identify different subdivisions and cell subpopulations of the central (EAe) and medial (EAme) extended amygdala in zebra finches, comparable to many of those described in mice (García-López et al. 2008; Waclaw et al. 2010; Carney et al. 2010; Bupesh et al. 2011a, b) and chickens (Abellán and Medina 2009; Vicario et al. 2014, 2015). Importantly, we have unequivocally identified the central and medial nuclei of the amygdala in zebra finches (Fig. 13), comparable to those described in other vertebrates (Moreno and González 2006; Martínez-García et al. 2007; Abellán and Medina 2009; Medina et al. 2011; Moreno et al. 2010, 2012a, b; Abellán et al. 2013; Vicario et al. 2014, 2015). This is discussed below in separate sections.

Many of the riboprobes for the hybridizations in zebra finches done in this study were based on the corresponding chicken genes. As explained in the Results section, this was done only with those genes found to be highly similar between chicken and zebra finch (sequence similarity higher to 90 %; see Table 2). The genes meeting this criterium were cIslet1, cPax6, cNkx2.1, cLhx6, cLhx9, cpENK, and cSOM (cSST). For all of these genes, the expression patterns visualized when using the antisense riboprobe in zebra finch brains were identical to those described in chicken (Abellán and Medina 2009; Vicario et al. 2014, 2015). In contrast, no signal was observed when using the sense riboprobe in parallel sections of zebra finch brains (Fig. 1 shows examples of sense versus antisense for cIslet1, cPax6, cNkx2.1, and cpENK), supporting the specificity of the signal obtained when using the antisense ribroprobes. Regarding cLhx6 and cLhx9, both showed very high similarity between chicken and zebra finch (close to 95 % or higher; Table 2). This, together with the facts that the antisense riboprobes of these two genes produced expression patterns in zebra finch identical to those found in chicken (Abellán and Medina 2009; Abellán et al. 2009), and identical to the expression patterns of the orthologous zebra finch genes (Chen et al. 2013) made us think that both are reliable for studies in zebra finches.

## Central extended amygdala (EAce)

The EAce is a subpallial cell corridor encompassing the intercalated cells, the central amygdala and BSTL (Alheid and Heimer 1988; de Olmos et al. 2004), and is involved in fear/anxiety responses and reward in different vertebrates (Martínez-García et al. 2007), aspects also relevant for modulating social behavior (Moore and Isen 1990). This structure has been recently redefined in mice and chickens based on developmental data (mouse: García-López et al. 2008; Bupesh et al. 2011b; chicken: Vicario et al. 2014, 2015). Based on the embryonic origin and genetic profile of its neurons, the EAce of mice and chickens appear to include several cell corridors derived from the dorsal striatal division (LGEd/Std, expressing Pax6 and perhaps proenkephalin), the ventral striatal division (LGEv/Stv, expressing Islet1, some of which may be those later expressing corticotropin-related factor or CRF), or the pallidal domain (MGE/Pa, expressing Nkx2.1, and some also somatostatin) (Bupesh et al. 2011b; Vicario et al. 2014; also discussed in Vicario et al. 2015). Data in mammals suggest that each of these three major neuron subpopulations of EAce may relate to a different functional pathway, modulating different aspects of the fear/anxiety response, motivation and pain (Bupesh et al. 2011b; Vicario et al. 2014; also discussed in Vicario et al. 2015).^1^ In mice, cells with dorsal striatal origin (expressing Pax6 and/or FoxP2, as well as pENK) tend to concentrate in the intercalated cells and the capsular subdivision of the central amygdala, while cells with ventral striatal origin accumulate primarily in the lateral and medial subnuclei of the central amygdala (expressing Islet1, and some possibly CRF) (Bupesh et al. 2011b; Waclaw et al. 2010). In addition, the central amygdala also contains a subpopulation of immigrant neurons expressing somatostatin, which originate in the ventrocaudal pallidal domain (García-López et al. 2008; Bupesh et al. 2011b). On the other hand, the BSTL is composed primarily of pallidal cells, but also includes an important subpopulation of cells that emigrate tangentially from the striatal division (Bupesh et al. 2011b). Based on these features and location, similar subdivisions and cell populations were recently identified in chickens (Vicario et al. 2014, 2015). However, the data in chickens helped to improve our knowledge on: (a) the neuron subtypes of the EAce and their origin, providing evidence for at least a triple origin of the pENK cells of BSTL and other subdivisions (in Std, in a dorsoventral pallidal subdivision or Padv, and in PO; see discussion in Vicario et al. 2014); (b) the extension of some EAce subdivisions, with the intercalated amygdalar cells extending more dorsally than previously thought to include the striatal capsule and, possibly, a comparable area in mice (see below); and (c) the differences in the abundance of some cell subpopulations between mice and chicken EAce, such as the Pax6 cells of dorsal striatal origin, which are very abundant in chicken BSTL, but very scarce in mice BSTL.

Our results in the zebra finch helped to identify the components of the central extended amygdala, including intercalated, central amygdala and BSTL parts, which were located in topological locations comparable to those described in chicken, and were characterized by similar expression patterns of transcription factors and phenotypic markers. This suggests a common organization pattern in the EAce between these two avian groups. However, the relative size of the EAce subdivisions was different between zebra finch and chicken, as discussed further below. Moreover, our data in zebra finch helped to define better a putative cell migration from the prethalamic eminence into the EAce, which led to identify extratelencephalic components in several of the subdivisions. The subdivisions found in zebra finch include: (1) Laterally: the intercalated-like cells (including the StC, located dorsally, and ventral ITC-like cell patches), the capsular central amygdala (CeC), the oval central nucleus (Ceov), the peri-INP, the perioval zone (pOv), and the rostral SpA (SpAr). (2) Medially, the BSTLd, which includes at least four subdivisions: medial, intermediate, lateral, and extratelencephalic. The EAce subdivisions of zebra finches were already visible at prehatching stages (St45, St46), the earliest we analyzed. At these prehatching stages, the zebra finch brain still showed a relatively immature aspect, comparable to E12-E14 of chicken (compare Fig. 2 in this study for zebra finch with Fig. 3 of Vicario et al. 2014, and Figs. 2–3 of Vicario et al. 2015, for chicken). This may be related to the differences in development of zebra finches and chickens: zebra finches are altricial (with delayed development, requiring nourishment after hatching), while chickens are precocial (Starck and Ricklefs 1998).

At pre-hatching stages, we identified a StC (dorsally) and the ventral intercalated-like patches (ITCv) with topological location and genetic profile similar to those in the chicken (Vicario et al. 2014, 2015). In both chicken and finch, the ITC-like cells along the StC and ventral patches appear distributed along the radial axis of the dorsal striatal division, as inferred from comparison to the radial glial fiber disposition seen in the chicken (Vicario et al. 2015) and the canary (Álvarez-Buylla et al. 1988). Both the StC and the ITCv were rich in cells expressing Pax6 and proenkephalin (pENK). As in chicken (Puelles et al. 2007; Abellán and Medina 2009; Vicario et al. 2014, 2015), the StC in zebra finch and other birds (vocal learners and non-vocal learners) includes cell clusters or patches expressing FoxP2 (Haesler et al. 2004). Such clusters expressing FoxP2 are also present along the external margin of the striatum in murines (rat: Takahashi et al. 2003; mouse: Campbell et al. 2009; Allen Developing Brain Atlas; called the lateral stripe of the striatum). The avian StC appears by deduced origin (in the dorsal striatal domain), position and molecular features directly comparable to the lateral stripe of the murine striatum (Kaoru et al. 2010). Since in mammals and birds the FoxP2 cells of the lateral stripe/StC are continuous with those of the intercalated amygdalar cells in mammals (Takahashi et al. 2003, 2008; Campbell et al. 2009; Kaoru et al. 2010), and with similar cell patches interposed between the arcopallium (part of the avian pallial amygdala) and the CeC in chicken (Vicario et al. 2014, 2015), we propose that both may represent dorsal and ventral aspects of the intercalated cell system of the amygdala. In our material of FoxP2 in zebra finches we could also observe the continuity between the cells of StC and those in the ventral intercalated-like patches (Fig. 10b, c). Nevertheless, in murines, only part of the FoxP2 of the intercalated amygdala expresses Pax6 at postnatal day 7 (Kaoru et al. 2010). However, Pax6 is strongly expressed along the whole radial division of the dorsal lateral ganglionic eminence at early stages in mice (E13.5), and continues showing moderate to strong expression in all ITC subdivisions at prenatal stages (Bupesh et al. 2011b). Therefore, one possibility that needs be further investigated is whether all FoxP2 cells of murine ITC derive from Pax6-lineage cells, even if some of them downregulate Pax6 expression later.

In addition, we identified a CeC comparable in position and genetic profile to that of mice and chickens (Bupesh et al. 2011b; Vicario et al. 2014). However, the CeC of zebra finch appeared to be smaller and less well defined than that in chicken. It is unclear if this is a difference between species, or may also be due to the age or the sectioning plane. We think that the age is unlikely to be the cause of the relatively small CeC seen in zebra finches, since the CeC develops quite early (seen from E9 in chicken; Vicario et al. 2014). The sectioning plane is also unlikely to contribute to the difference since at prehatching stages (St. 45, St. 46) we have brain series of zebra finches sectioned at a plane comparable to the frontal plane employed in chicken at a comparable age (i.e., about E12–E14) (for example, see Fig. 2 of this study).

Like in chicken (Vicario et al. 2015), the Pax6 and enkephalinergic cell subpopulations of the StC, ITCv, and CeC may come from the Std domain, and their radial continuity with this domain agrees with this proposal. However, it appears that ITCv/CeC may additionally have a minor extratelencephalic cell subpopulation; in particular a subtype of Pax6 cells apparently coming from the prethalamic eminence (EMT), as also may happen in chicken (discussed above; see also Abellán and Medina 2009) and perhaps mice (Bupesh et al. 2011b). Employing zebra finch as a model allowed us to better observe and follow the putative migration trajectory of these EMT-derived cells into the caudal telencephalon, since these cells were not only observed with Pax6, but also with Lhx5 (see the remarkably strong expression of such EMT-derived cells in Fig. 6a–c of this study). The EMT was also observed to produce some Lhx5 cells for the telencephalon in mice and chicken (Abellán et al. 2010), but it was thought that these cells partly represented a subpopulation of Cajal-Retzius cells and partly a subpopulation of the medial extended amygdala. It is unclear whether the Pax6 and Lhx5 cells derived from EMT represent the same (at least partially) or different subpopulations. Based on Pax6 and Lhx5, some EMT-derived cells appear to tangentially invade different parts of the EAce, including the ITCv, CeC and the BSTL (see below). We have proposed to use the suffix –e-, meaning extratelencephalic, for all the structures we believe contain these minor extratelencephalic cell populations. In the particular case of the central capsule, this extratelencephalic component is named CeCe (Fig. 6b, c). We believe this nomenclature clarifies the different components of each major subdivision of the EAce, and agrees with the concept of the extended amygdala as a mosaic composed by different cell corridors populated by cells with a different embryonic origin (see Medina and Abellán 2012, and Vicario et al. 2014, 2015, for a better comprehension). In any case, it remains unknown if all the putative EMT-derived cell subpopulations we have seen in the zebra finch EAce are also present in chicken and other vertebrates.

Within the central nucleus, in addition to CeC we identified two other subdivisions rich in Islet1 expressing cells (the oval central nucleus or Ceov) or in both Pax6 expressing and Islet1 expressing cells (peri-INP or pINP), comparable to similar subdivisions found in chicken (Vicario et al. 2014, 2015). Both were visible from prehatching stages in zebra finch, but the Ceov was remarkable since it appeared as a huge cell mass, rich in Islet1-expressing cells. Whereas the zebra finch CeC (laterally adjacent to Ceov) appears smaller than that of chicken (see above), the Ceov looks larger than its chicken counterpart. More studies are needed to investigate whether this is due to species differences and, if so, analyze the functional and behavioral consequences. As is the case in chicken (Vicario et al. 2014, 2015), the finch pINP is located within the radial ventral striatal domain, while the Ceov in zebra finch is located within the pallidal radial domain (characterized by the vz/svz expression of Nkx2.1), and this may be due to a tangential migration of the Islet1 cells from their site of origin in the Stv ventralwards, similar to what happens in chicken (Vicario et al. 2014, 2015).

As discussed for the chicken (Vicario et al. 2014, 2015), the Ceov together with the pINP (located above the Ceov) appear comparable to the CeL/CeM of mice. Like in the chicken (Vicario et al. 2014, 2015), the pINP of zebra finches contains a mixture of Islet1- and Pax6-expressing cells, which likely originate in the Stv and Std domains, respectively. In chicken, both Ceov and pINP contain a subpopulation of CRF cells, which is a typical cell subpopulation of rodent CeL, known to project to the BSTL and the brainstem (Moga and Gray 1985, Moga et al. 1989) and be involved in anxiety (Davis et al. 2010). In mice and chickens, it has been proposed that such CRF cells originate in Stv and express Islet1 (Bupesh et al. 2011b; Vicario et al. 2014, 2015). It would be interesting to study whether a similar cell subpopulation is present in the zebra finch Ceov/pINP.

As described in chicken (Vicario et al. 2014), the Pov is another subdivision of EAce also in the zebra finch, and appears as a cell corridor rich in enkephalinergic cells extending radially from the Padv into the medial part of the mantle. This cell corridor has been compared to the sublenticular part of the EAce of mice, which also contains pENK expressing cells (Bupesh et al. 2011b; discussed in Vicario et al. 2014). In chicken, this cell corridor extends laterally from the BSTLd, and interposes between Ceov and pINP (Vicario et al. 2014). However, in the zebra finch, the Pov is observed caudal to the Ceov, although it also extends laterally from BSTLd. Thanks to the oblique section plane used in adult zebra finches, it was possible to observe that this cell corridor extends above the medial extended amygdala, which includes the medial nucleus located caudolaterally in the telencephalon (see Fig. 4e). The Pov cell corridor appears to run parallel to the ventroamygdalofugal tract (vaf) but dorsal to it. Thus, the Pov is seen in a partially different position when comparing zebra finch and chicken (Vicario et al. 2014), although in both species it is a pENK-positive cell corridor derived from the Pad and medially related to the BSTLd. The variation between species affects the lateral aspect of Pov, and may be due to the different growth and divergence of the telencephalon in both species. However, to understand better the differences observed between species, it would be very useful to study the enkephalinergic expression in chicken using a sectioning plane similar to that employed in adult zebra finch (i.e., close to horizontal). The Pov also appears to have a cell subtype expressing Lhx5 with extratelencephalic (EMT) origin, named Pove (see Fig. 6c).

At rostral levels of EAce, we also identified the SpAr as an area containing different cell subpopulations, such as Islet1 (of putative striatal origin) and Nkx2.1 (of putative pallidal origin). This area was located in a topological location similar to that in the chicken (i.e., in the dorsal pallidal domain), and also had a similar cell composition (Vicario et al. 2014). In chicken, this area is densely innervated by CGRP fibers (Martínez-García et al. 2008), and based on this and its location it appears comparable to the reptilian striatoamygdaloid area (SAT) and perhaps the mammalian interstitial nucleus of the posterior limb of the anterior commissure (IPAC), both part of the EAce (discussed in Vicario et al. 2014).

The area defined as SpAr in chicken and zebra finch appears to overlap with the caudolateral pole of the accumbens shell, as defined by Abellán and Medina (2009) based on Lmo4 expression, and by Csillag and colleagues based on connectivity patterns (Mezey and Csillag 2002; Bálint and Csillag 2007; Bálint et al. 2011). However, according to Abellán and Medina (2009), the accumbens shell is a striatal structure, while the SpAr is located in the pallidal territory (as seen in the present study). The SpAr is characterized by a dense CGRP innervation (Lanuza et al. 2000; Martínez-García et al. 2008), which helps to distinguish it from the accumbens shell. It would be important to study further the relation between SpAr and the accumbens shell to know whether they represent the same or different structures. The accumbens shell has been considered part of the EAce in mammals (Alheid et al. 1995; de Olmos et al. 2004). The projections of the region encompassing the avian SpAr and accumbens shell to the hypothalamus and brainstem are very similar to those of the BSTL (Bálint et al. 2011, 2014). Therefore, it is reasonable to think that all of them may belong to the same functional system.

Regarding the medial part of the EAce or BSTLd, this nucleus was also identified in zebra finch as a pallidal structure, rich in Nkx2.1 and located in the radial pallidal domain, similarly to that of chicken (Abellán and Medina 2009; Vicario et al. 2014). Both in chicken and finch, the BSTLd also includes subpopulations of Pax6 and Islet1 expressing cells, and in chicken these have been shown to emigrate tangentially from either Std or Stv, respectively (Vicario et al. 2015). This is likely the case in zebra finch. Both in chicken and finch, the BSTLd also includes subpopulations of pENK cells (Vicario et al. 2014; present results). As suggested in chicken (Vicario et al. 2014, 2015), the pENK cells of BSTLd may have three different origins: part may emigrate from the striatal domain (as those with Pax6 and Islet1), part may originate in the Padv, and a few may come from the PO. Nevertheless, this suggestion needs to be checked by fate mapping studies. In chicken, we identified three subdivisions within the BSTLd, medial, intermediate and lateral, each characterized by a different combination and organization of the above-mentioned cells (Vicario et al. 2014). These three subdivisions were also observed in the zebra finch starting at prehatching stages, with only slight differences with those of chicken. In the zebra finch, the BSTLdm contained pENK cells and a compact cell stratum expressing Islet1, similarly to that in chicken (Vicario et al. 2014). However, the Islet1 expression in the finch BSTLdm is rather weak compared to that seen in the BSTLdm of chicken (Vicario et al. 2014), and this may be due either to the age (its expression rapidly declined after hatching in zebra finches) or the fact that we were using riboprobes based on the chicken gene sequence. However, we could easily identifiy the BSTLdi, rich in Pax6 and pENK expressing cells, resembling that of chicken (Vicario et al. 2014), although this division was more remarkable in zebra finch. The pENK and the Pax6 cells of the BSTLdi form a continuum with those in the striatum, suggesting that these cells immigrate from the striatal domain (as shown experimentally in chicken for the Pax6 cells; Vicario et al. 2015). Lateral to the latter subdivision, we identified the BSTLdl, containing dispersed subpopulations of Islet1- and Pax6-expressing cells. In addition, in the zebra finch we identified a small extratelencephalic subdivision in the BSTLd, the BSTLde. It is populated by Pax6-expressing cells, but also by Lhx5 expressing cells. The Pax6 cells of BSTLde may immigrate from the prethalamic eminence (EMT). In chicken, we also proposed that a few Pax6 cells of the caudolateral BSTLd may immigrate from EMT (discussed above and Vicario et al. 2014, 2015; see also Puelles et al. 2000), and such subpopulation of EMT-derived cells also appear to be present in the BST/BSTL of other amniote vertebrates, such as the turtle (Moreno et al. 2010, 2012a) and the mice (Bupesh et al. 2011b), but this extratelencepahlic subpopulation is not present in unamniotes as the frog (Moreno et al. 2012b). On the other hand, the transcription factor Lhx5 is expressed by preoptic (PO), hypothalamic (SPV) and diencephalic (EMT) derivatives, all of which produce cells destined for the medial extended amygdala (see also Abellán et al. 2010 and below). However, the expression of Lhx5 by these three derivatives is much stronger in the zebra finch than in chicken, and this has allowed the identification of cells from at least the EMT and PO reaching not only the BSTM (see below, and Abellán et al. 2010), but also the BSTL.

One important difference between the subpallial and extratelencephalic components of the BSTLd and other parts of the EAce is that while the former are constituted by GABAergic cells, the latter are formed by glutamatergic neurons (Abellán et al. 2013; also discussion in Vicario et al. 2014, 2015). This raises questions on the connections and functions of these different cells. Recently, the BSTL of mammals was reported to include both GABAergic (the majority, about 90 % of the projecting cells) and glutamatergic projections to the ventral tegmental area (VTA) (Kudo et al. 2012), and their stimulation led to either reward and anxiolysis (the GABAergic projections) or aversion and anxiogenesis (the glutamatergic projections) (Jennings et al. 2013a). Thus, these different cells produce opposite effects on motivation. It is likely that the GABAergic VTA projecting cells are subpallial in origin, while the glutamatergic cells are extratelencephalic, perhaps with EMT origin. In different vertebrates, the EMT is known to also express the transcription factor Tbr1 (Puelles et al. 2000, 2004; Medina et al. 2004; Moreno et al. 2010), involved in the differentiation of glutamatergic neurons (Hevner et al. 2001). Since the BSTL also projects to the VTA in birds (Atoji et al. 2006), it is possible that GABAergic (major) and glutamatergic (minor) projections are also present.

Our results in chicken (Vicario et al. 2014, 2015) and zebra finch (present results) point to the existence of several subpopulations of GABAergic cells in the BSTLd and other parts of the EAce. This may also be similar in mice (Bupesh et al. 2011b), and raises questions about the specific projections and functions of each different GABAergic cell subtype. For example, are the GABA BSTL-VTA projecting cells (Kudo et al. 2012; Jennings et al. 2013a; as noted above, these are involved in reward and anxiolysis) the same as those projecting to the lateral hypothalamus, which are involved in motivational regulation of feeding (Jennings et al. 2013b)? Are these the same as those projecting to the periaqueductal gray (PAG) or other brainstem targets? If they do project to the same targets, they may end on different cells and/or use different receptors, and may regulate different aspects of motivation, fear and nociception. It appears that some of the cells with different origin can be distinguished by their expression of neuropeptides. For example, CRF cells of the central amygdala may come from Stv/LGEv, pENK cells may come from Std/LGEd, and SOM cells may come from Pavc/MGEvc, and this may be partially similar for these cells in the BSTL/BSTLd (Bupesh et al. 2011b; Vicario et al. 2014, 2015). However, for the case of the SOM cells and the ENK cells, the situation is more complicated (see discussion above). For example, our data in chicken and zebra finch suggest that ENK cells of the BSTLd may have several origins, in the striatal domain (perhaps including both Std and Stv), in the pallidal domain (Padv) and a few ENK cells also come from the preoptic region. Therefore, it is important to find additional markers that help to discriminate between EAce neurons with different origins, for then carrying out a re-evaluation of the projections of these different cells, as a first step to understand their function.

## Medial extended amygdala (EAme)

In mammals, the EAme includes the medial amygdala and the BSTM (Alheid and Heimer 1988). Recent developmental studies in mice have revealed the complex cellular composition of the medial amygdala and BSTM, with cells from multiple origins (García-López et al. 2008; Medina et al. 2011; Abellán et al. 2013). Although it is primarily a subpallial cell corridor, it contains minor subpopulations of cells from the pallium, and minor to moderate subpopulations of cells coming from outside the telencephalon (reviewed by Abellán et al. 2013). Genetic and experimental fate mappings, including tracing of specific cell lineages using the Cre-loxP system (Xu et al. 2008; Hirata et al. 2009; Carney et al. 2010; Puelles et al. 2016), and tracing of pools of progenitor cells by in utero electroporation (Soma et al. 2009; García-Moreno et al. 2010) or by migration assays in tissue culture (Bupesh et al. 2011a, b) have been used for elucidating the cell origin of the different components of the mammalian medial extended amygdala. These fate mapping studies have revealed that the medial amygdala contains cells derived from the ventral pallium (expressing Lhx9), the ventrocaudal pallidum (MGEvc, expressing Nkx2.1 and Lhx6), commissural preoptic area (POC, expressing Shh and Dbx1), and the supraopto-paraventricular hypothalamic domain (SPV, expressing Otp and Lhx5) (Hirata et al. 2009; Soma et al. 2009; Carney et al. 2010;  García-Moreno et al. 2010; Bupesh et al. 2011a; Puelles et al. 2016). On the other hand, the BSTM appears to include cells from the ventrocaudal pallidum (MGEvc, expressing Nkx2.1 and Lhx6), commissural preoptic area (POC, expressing Shh), the supraopto-paraventricular hypothalamic domain (SPV, expressing Otp and Lhx5) and possibly the prethalamic eminence (EMT, expressing Pax6) (Puelles et al. 2000; Hirata et al. 2009; Soma et al. 2009; Carney et al. 2010; Bupesh et al. 2011a, b). It appears that each of these different cells is involved in a specific functional pathway, as shown for the Lhx6 cells of the medial amygdala and BSTM derived from MGEvc, which are involved in sexual behavior (Medina et al. 2011; Abellán et al. 2013; see also Sokolowski and Corbin 2012). In addition to the functional implications, this knowledge sets the basis for comparing with other vertebrates and for trying to investigate the evolutionary origin of each specific cell type.

The medial extended amygdala, including nucleus taeniae (as the putative homolog of the medial amygdala) and the BSTM, was previously identified in different birds, including galliformes (quail, chicken), columbiformes (pigeon), psittaciformes (budgerigars) and oscine passeriformes (songbirds) (Aste et al. 1998; Jurkevich et al. 1999; Roberts et al. 2002; Reiner et al. 2004a, b; Yamamoto et al. 2005). This was based on their content of cells expressing vasotocin and estrogen receptors, their projections to the preoptic region and medial hypothalamus, and their role in sexual behavior (reviewed in Reiner et al. 2004a; Medina et al. 2011; Kuenzel et al. 2011). However, in the Avian Brain Nomenclature Forum it became evident that only a rostromedial part of the so-called nucleus taeniae may be comparable to the medial amygdala of other vertebrates, which is primarily a subpallial structure (Reiner et al. 2004a). Moreover, the nucleus taeniae identified in zebra finches (Ikebuchi et al. 2013) does not appear to correspond to the subpallial nucleus taeniae recognized by the nomenclature forum as the likely homolog of the mammalian medial amygdala (Reiner et al. 2004a), since such nucleus in zebra finches is located within the arcopallium. A similar situation applies to the nucleus taeniae identified in budgerigars (Roberts et al. 2002). Moreover, nucleus taeniae of budgerigars is rich in parvalbumin cells (Roberts et al. 2002), but that type of cells is only found in the pallial amygdala, but not in the subpallial amygdala in mammals (Kemppainen and Pitkänen 2000). Thus, nucleus taeniae of zebra finches and budgerigars is a pallial nucleus that is not comparable to the subpallial part of the mammalian medial amygdala, although we cannot exclude that it may be comparable to the Lhx9-expressing cell subpopulation of ventral pallial origin that is found within the medial amygdala in mice (García-López et al. 2008; Bupesh et al. 2011a; see below).

Recently, Abellán and Medina (2009) used a battery of region-specific transcription factors and other regulatory proteins for trying to identify the components of the EAme in chicken embryos. The comparison with mammals was based on topological criteria, apparent embryonic origin and genetic profile of the cells (Abellán and Medina 2009). On the basis of these criteria, Abellán and Medina (2009) identified the subpallial medial amygdala (MeAs), containing cells with expression of Nkx2.1, Lhx6 and Shh, likely derived from the pallidal (Pavc) and POC domains. This structure corresponds to what Reiner et al. (2004a) (Avian Brain Nomenclature Forum) called the subpallial division of nucleus taeniae. Since this name is also used to refer to a pallial nucleus (Puelles et al. 2007; Ikebuchi et al. 2009, 2013), to avoid confusion, in the present study we adopted the nomenclature of Abellán and Medina (2009) regarding the avian medial extended amygdalar structures (see next paragraph). In particular, we are using the term subpallial medial amygdala (MeAs) or just medial amygdala (MeA) to refer to the nucleus rich in Nkx2.1, Lhx6 and Shh expressing cells, which are comparable to the pallidal and preoptic cellular components of the medial amygdala of mammals (Abellán and Medina 2009). In addition to the subpallial cells, it appears that this nucleus includes minor subpopulations of immigrant cells from the ventral pallium (expressing Lhx9) (Abellán et al. 2009, 2013), from the hypothalamic SPV (expressing Otp, Bardet et al. 2008), and from the prethalamic eminence (EMT, expressing Pax6 and/or Lhx5; Puelles et al. 2000; Abellán et al. 2010), resembling the situation in mice (Medina et al. 2011; Abellán et al. 2010, 2013). In reptiles (turtles and lizards), the medial amygdala contains at least the subpallial cells expressing Nkx2.1 (from the pallidum), and the subpopulations of immigrant cells expressing Lhx9 (from the ventral pallium), Otp (from the hypothalamus) and Pax6 (from the EMT) (Moreno et al. 2010; Abellán et al. 2013). However, there are no data on Shh expression in reptiles and this, together with the lack of fate mapping data, impedes knowing whether the reptilian medial amygdala also includes a POC-derived cell subpopulation. Thus, most of the components of the medial amygdala found in mice and chickens may have been present in the amniote ancestor.

By analyzing the expression patterns of some of the genes expressed in the medial extended amygdala of mice (García-López et al. 2008; Bupesh et al. 2011a) and chicken (Abellán and Medina 2009), we have identified in the caudolateral telencephalon of the zebra finch a nucleus that resembles the medial nucleus of the amygdala. It coincides in location and the genetic profile of its cells with that of mice and chicken (Abellán and Medina 2009; Abellán et al. 2013), and with the subpallial component of the nucleus taeniae defined by the Avian Brain Nomenclature Forum (Reiner et al. 2004a). In contrast, the nucleus taeniae identified in zebra finches (Ikebuchi et al. 2013) appears to develop in the arcopallial region, a territory rich in expression of the pallial marker Lhx9, but poor in subpallial (pallidal) marker Lhx6 (Medina and Abellán 2009). This discards this nucleus as a homolog of the subpallial medial amygdala although, as noted above, we cannot discard that nucleus taeniae of zebra finches may be comparable to the Lhx9-expressing cell subpopulation of ventral pallial origin that is found within the medial amygdala in mice (García-López et al. 2008; Bupesh et al. 2011a). The subpallial medial amygdala identified here in zebra finches contains a subdivision rich in cells expressing Nkx2.1 and Lhx6 apparently derived from the pallidum, but also contains subdomains with cells from other origins: (1) many Lhx5-expressing cells, which concentrate in a subdomain medially adjacent to the pallidal part of the medial amygdala; these cells may derive from preoptic and extratelencephalic domains (as explained below). (2) A very small subpopulation of Lhx9–expressing cells, which appear to derive from the adjacent ventrolateral caudal pallium (arcopallium).

The minor cell subpopulation of Lhx9 cells of MeA with apparent ventral pallial origin has also been found in the medial amygdala of mice (Bupesh et al. 2011a), chicken (Abellán and Medina 2009) and lizard (Abellán et al. 2013). In mice and other vertebrates such Lhx9 cell subpopulation of ventral pallial origin migrates tangentially into the subpallium, neighboring or mixing with medial amygdalar cells of subpallial origin (García-López et al. 2008; Bupesh et al. 2011a; Abellán et al. 2013). However, it is possible that part of such cells remain behind, within the pallium, in some vertebrates as the finches. Regarding the Lhx5 cells of the medial amygdala, based on our observations in zebra finch, these cells could come from the PO, EMT, or SPV (as discussed above). In mice, at least part of the Lhx5 cells of the medial amygdala coexpress Otp (García-Moreno et al. 2010), and are thought to originate in the SPV (García-Moreno et al. 2010). In chicken, turtle and lizard, the hypothalamic SPV also produces Otp cells for the medial amygdala (Bardet et al. 2008; Moreno et al. 2010; Abellán et al. 2013). However, although Lhx5 cells are also present in the medial amygdala of chicken, we could not see these cells migrating from the SPV, but they appear to immigrate from the EMT and possibly the PO (Abellán et al. 2010). The PO and the EMT also appear to produce Lhx5 cells for the medial amygdala in mice (Abellán et al. 2010). Thus, perhaps the medial amygdala in mice and birds contains three different subpopulations of Lhx5 cells (from PO, SPV or EMT), but there are variations between species in the abundance of each cell type. Fate mapping experiments are needed to verify, modify or discard this proposal, and understand better the differences between species. These findings also open a new venue for investigating the connections and function of each cell type.

The medial amygdalar nucleus of zebra finch is also rich in cells containing somatostatin (SOM) and enkephalin (pENK), which appear to come from the Pavc and Padv, respectively. This is based on the observations discussed above for the EAce. A cell corridor of pENK cells is seen extending from the dorsoventral pallidal progenitor domain, Padv, laterally through the Pov. Some of these pENK may populate the medial amygdalar nucleus, although other pENK cells of the medial amygdala may originate elsewhere. This may be similar in chicken (unpublished observations). Regarding the SOM cells, as in mammals and chicken, in the zebra finch the SOM-expressing cells of the extended amygdala may originate in the Pavc, since a stream of such cells is visible extending from this domain laterally until reaching the medial amygdala; this stream of SOM cells is parallel but ventral to that of the ENK-expressing cells mentioned before. Laterally, the SOM cells aggregate in the medial amygdalar nucleus, in a more ventral location with respect to the enkephalinergic cell subpopulation (compare Fig. 4c, e). Both pENK and SOM cells are located in the pallidal component of the medial amygdala, overlapping those expressing Lhx6.

Regarding the BSTM, this nucleus was slightly redefined in chicken to include also part of the previous BSTL (Abellán and Medina 2008, 2009; initial definition by Aste et al. 1998), and was proposed to include cells derived from the Pavc (Nkx2.1 and Lhx6), the POC (Shh and Lhx7), the hypothalamic SPV (Otp) and the EMT (Pax6 and Lhx5) (Puelles et al. 2000; Abellán and Medina 2008, 2009; Abellán et al. 2009, 2010; Bardet et al. 2008), resembling the BSTM of mice (Abellán et al. 2010, 2013). The exact location of this nucleus in reptiles is unclear, but possibly is more caudal than the BST (which mostly resembles the lateral BST of mammals and birds; Moreno et al. 2012b), as identified in lizards by Abellán et al. (2013), in a location resembling its position in chicken.

In zebra finch, the BSTM appears to include at least three parallel subdomains of cells with different genetic profile and apparent origin, as follows. From medial to lateral, it can be divided into: (1) A preoptic component of the BSTM, BSTMpo, which is seen as a stream of cells crossing from ventral to dorsal the anterior commissure. These cells express Lhx5 and likely come from the commissural preoptic subdivision, as in mice (Bupesh et al. 2011a) and chicken (Abellán and Medina 2009; Abellán et al. 2010). This is the component of the BSTM located in the more medial zone, close to third ventricle and its apparent progenitor domain. In chicken and zebra finch, this subdivision of the BSTM is rich in cells expressing Islet1 (Vicario et al. 2014, 2015). In chicken, the BSTMpo subdivision is rich in expression of Lhx7, as other derivatives of the preoptic area (Abellán and Medina 2008, 2009), but we have no data on Lhx7 in zebra finches. In zebra finch this subdivision also includes a subpopulation of Lhx6-expressing cells, and is also populated by somatostatinergic cells. (2) A pallidal component of the BSTM, BSTMpa, located just laterally to the preoptic cell stream or subdomain. The pallidal BSTM could be identified as a cell corridor rich in Lhx6-expressing cells, but poor in Islet1, which is also present in chicken (Abellán and Medina 2009) and mice (García-López et al. 2008). These cells appeared to span from the pallidal domain (possibly the Pavc) to populate lateral and ventral zones of the mantle. This division includes subpopulations of somatostatinergic cells, but was generally poor in cells expressing Lhx5 and pENK. We could see this pallidal subdivision with Lhx6, extending ventrally from Pavc, from early posthatching stages (PHD4; we did not have Lhx6 at earlier stages), and was still visible at PHD25 as a distinct cell corridor of SOM cells. (3) A hypothalamic component, rich in cells with expression of Lhx5, but poor in Lhx6 and Islet1. We called this division the hypothalamic BSTM, BSTMh. It consists of cells that form a continuum with those in the SPV, suggesting that this is the origin of such cells, and from here they migrate tangentially in a dorsal direction. The BSTMh has been identified in mice and chickens as a cell corridor rich in Otp and/or Lhx5, extending from the hypothalamic SPV to the medial amygdala (Bardet et al. 2008; Abellán et al. 2010, 2013; García-Moreno et al. 2010; Bupesh et al. 2011a). In finch, the hypothalamic subdivision of the BSTM also contains a subpopulation of enkephalinergic cells. Notably, our data show that the hypothalamic component of the BSTM is also populated by mesotocinergic cells (containing mesotocin, MT, the homolog of oxytocin, Goodson et al. 2012). Because of their continuity with those in the paraventricular hypothalamic nucleus, these MT cells may be produced in the SPV hypothalamic domain, as suggested in chicken by Arnold-Aldea and Sterritt (1996). The BSTM also contains vasotocin neurons (AVT, homolog of vasopressin; Goodson et al. 2012), which may also originate in the SPV (Abellán et al. 2013). In birds, the AVT cells are distributed in two separate cell corridors (galliformes: Aste et al. 1998; passerines: Panzica et al. 1999), one medial (with magnocellular and parvocellular cells) which appears to partly overlap and/or neighbor caudally our preoptic BSTM, and another one lateral (with magnocellular cells) which appears to correspond to our hypothalamic BSTM. These two cell corridors are also visible by expression of vasotocin receptors in zebra finches and other songbirds (Leung et al. 2011; Grozhik et al. 2014). In these studies, only the medial corridor is called BSTM, but the lateral one has been suggested to be part of this nucleus (Fig. 5 in Leung et al. 2011).

Mesotocin and vasotocin cells, including those of the BSTM, play a very important role in some aspects of social behavior, including gregariousness, pair-bonding and aggression (Goodson et al. 2009, 2012; Kelly and Goodson 2013, 2014). The role of oxytocin and vasopressin is evolutionarily highly conserved, and these peptides act in a sex and species-specific manner (Young and Wang 2004; Carter et al. 2008; Donaldson and Young 2008). In adult zebra finches, many studies have revealed different effects of vasotocin and oxytocin/mesotocin, such as promoting preferences for large groups rather than small (Goodson et al. 2009; Kelly et al. 2011; Kelly and Goodson 2013), for familiar mates instead of unknown comrades (Goodson et al. 2009), or the effect on pair-bonding (Kingsbury and Goodson 2014). These social abilities appear to be facilitated by a general anxiolytic effect (Kelly et al. 2011). Importantly, the BSTM is one of the forebrain structures implicated in these behaviors (Goodson et al. 2012), and our data suggests that its hypothalamic subdomain may be particularly involved.

In conclusion, the BSTM of zebra finch have at least three subdomains primarily populated with cells of different embryonic origin: preoptic, pallidal and hypothalamic. Nevertheless, it appears that some intermingling occurs, as some of the vasotocin cells of putative hypothalamic origin area may partly overlap the preoptic subdomain (as noted above). In addition to these, the BSTM also contains EMT derived cells, but it is unknown whether they overlap with the other cells, or occupy a different space within the nucleus. The BSTM in chicken (Abellán and Medina 2009; unpublished observations) and mice (García-López et al. 2008; Bupesh et al. 2011a) are also formed by parallel cell corridors with distinct genetic profile and embryonic origin, and this may be a common feature in amniotes (although data in reptiles are needed).

As suggested in mice and chickens, the cells with different origin of BSTM may be enrolled in different functional pathways, and modulate different aspects of social behavior (Abellán et al. 2013). For example, the Lhx6 cells (with pallidal origin) may be involved in sexual behavior (as in rat: Choi et al. 2005), while those with Otp (from the hypothalamus) may include the vasotocin/vasopressin and mesotocin/oxytocin cell subpopulation, involved in pair-bonding and affiliation (Abellán et al. 2013; Kelly and Goodson 2014). The roles of the cell subpopulations that originate in the preoptic region and the EMT are unknown. As noted above, the medial amygdala also includes cell subpopulations from the same origins as those in the BSTM (pallidal, preoptic, hypothalamic, prethalamic eminence), which may be enrolled in the same functional pathways as those in the BSTM (Abellán et al. 2013). In addition to these cells, the medial amygdala includes a minor subpopulation of Lhx9 cells of ventral pallial origin, whose connections and role are unknown (but see Abellán et al. 2013). In mammals, cells of different embryonic origin show a trend to segregate into different subdivisions in the posterior part of the medial amygdala, but they are mostly intermingled rostrally (García-López et al. 2008; Abellán et al. 2010). In chicken, these different cells were found to overlap, which led to the suggestion that this nucleus was comparable to the anterior part of the mammalian medial amygdala (Abellán and Medina 2009), in agreement with a previous proposal based on the type of olfactory input this nucleus receives (Yamamoto et al. 2005). In zebra finches, it appears that different cells occupy different subdomains of the medial amygdala: Lhx6- and Nkx2.1-expressing cells are located in the laterodorsal subdivision of this nucleus, whereas Lhx5-expressing cells are placed medioventrally and in the surface of the nucleus (present results). It is unclear whether the segregation pattern of cells with different origin found in the medial amygdala is ancestral or derived, and more studies including reptiles are needed.

## FoxP2 in the extended amygdala of zebra finch

We investigated the expression of FoxP2 in the different components of the extended amygdala, since alterations in the gene encoding this transcription factor have been associated with speech and language deficits in humans (reviewed by Marcus and Fisher 2003; Fisher and Scharff 2009, and French and Fisher 2014), and may contribute (not alone, but in combination with other genes) to the development of autism (Park et al. 2014), which implies not only deficits in communication but also in social skills (Bacon and Rappold 2012). Songbirds like zebra finches are excellent models for studying the role of FoxP2 in the brain, since they use vocalization (song) for social communication, and this transcription factor has also been shown to be required for song learning and social modulation of adult song (Scharff and Haesler 2005; Wohlgemuth et al. 2014). In mice, FoxP2 is expressed in the extended amygdala, including the intercalated cells and the medial amygdala (Campbell et al. 2009; Kaoru et al. 2010), but, other than that, the exact location of the expression within this mosaic-like complex structure is unknown. Here we have used the zebra finch to investigate the location of FoxP2, paying attention to the cell components with different embryonic origin and genetic profile of the EAce and EAme.

Our results show that several subnuclei within the EAce in zebra finches contain cells expressing FoxP2, although with variations in their density and abundance between subdivisions. Maybe this transcription factor, which is playing an important role in synaptic plasticity relevant for vocal learning (reviewed by Bolhuis et al. 2010), is somehow implicated in the synaptic plasticity underlying the mechanisms in which this structure is involved, such as acquisition, consolidation and expression of fear conditioning (Ciocchi et al. 2010; Durvaci et al. 2011), or in recall and extinction of fear memories in which the ITC are involved (Paré et al. 2004; Paré and Durvaci 2012), or contextual fear where BSTL is involved (Phelps and LeDoux 2005; Walker and Davis 2008; Durvaci et al. 2009). As in mice (Campbell et al. 2009; Kaoru et al. 2010), our data in zebra finch indicate that FoxP2 is particularly enriched in the proposed ITC-like cells, including the dorsal part (StC, see also Haesler et al. 2004) and the ventral intercalated-like patches (present results). The role of these cells in zebra finches and other birds are unknown. Many cells expressing FoxP2 are also observed in the pINP and in the BSTL, and more studies are needed for understanding what cell subtype of these subdivisions contains FoxP2, and the role of FoxP2 in such cells.

We have also found the presence of many FoxP2 expressing cells in the medial extended amygdala of zebra finch, including the medial amygdala and BSTM. In the BSTM, the FoxP2 cells appear to be more abundant in the preoptic, hypothalamic and EMT-derived cells, although some cells are also observed in the pallidal subdomian. Double-labeling experiments will be needed to know which specific cell types express FoxP2. In any case, it appears that this transcription factor is expressed in cells of different origins and may be playing a role in the plasticity of different functional pathways. Two of the cell types that may be expressing FoxP2 are the subpopulations of mesotocinergic (MT) and vasotocin (AVT) cells, probably coming from this hypothalamic SPV domain, which play a very important role in sex-specific gregariousness and pair-bonding (Kelly and Goodson 2014). It would be important to investigate whether this is so in mammals, which may help to understand the association of FoxP2 in the development of the social deficits observed in autism (Park et al. 2014).

## Abbreviations

3v: Third ventricle
i1: Tangentially oriented intermediate cell corridor rich in Pax6
A: Arcopallial amygdala
ac: Anterior commissure
al: Ansa lenticularis
APH: Parahippocampal area
BST: Bed nucleus of the stria terminalis
BSTL: Lateral part of BST
BSTLd: Dorsal division of BSTL
BSTLde: Extratelencephalic division of BSTLd
BSTLdi: Intermediate division of BSTLd
BSTLdl: Lateral division of BSTLd
BSTLdm: Medial division of BSTLd
BSTM: Medial division of BST
BSTMpa: Pallidal division of BSTM
BSTMpo: Preoptic division of BSTM
BSTMh: Hypothalamic division of BSTM
Ce: Central nucleus of the central extended amygdala
CeC: Capsular central amygdala (or capsular part of Ce)
CeCe: Extratelencephalic component of CeC
Ceov: Oval central nucleus (or oval division of Ce)
chp: Choroid plexus
CLSt: Caudolateral striatum
co: Chiasma opticum
csm: Corticoseptomesencephalic tract
DB: Diagonal band nuclei
DLA: Dorsolateral anterior nucleus of the thalamus
EAme: Medial extended amygdala
EMT: Prethalamic eminence
GP: Globus Pallidus
Hb: Habenula
Hbp: Basal hypothalamus, peduncular part, periventricular subdivision
Hy: Hypothalamus
IC: Intercalated zone of the prethalamus
IGL: Intergeniculate leaflet
INP: Intrapeduncular nucleus
ITC: Intercalated-like cell patches
ITCv: Ventral part of the ITC
lfb: Lateral forebrain bundle
LHy: Lateral hypothalamus
LPO: Lateral preoptic area
LSt: Lateral striatum
lv: Lateral ventricle
ME: Median eminence
MeA: Medial amygdala
mfb: Medial forebrain boundle
MG: Medial geniculate nucleus (or nucleus ovoidalis of the thalamus)
MP: Medial pallium
MPG: Medial zone of the pregeniculate nucleus
MSt: Medial striatum
ot: Optic tract
OvP: Oval prethalamic nucleus (or nucleus lateralis anterior)
Pa: Pallidal embryonic division
Pad: Dorsal part of Pa
Padd: Dorsal division of Pad (or dorsodorsal part of Pa)
Padv: Ventral division of Pad (or dorsoventral part of Pa)
Pavc: Ventrocaudal part of Pa
PG: Pregeniculate nucleus
pINP: Peri-INP area
PO: Preoptic area
POB: Basal or ventral part of PO
POC: Commisural part of PO
POM: Nucleus preopticus medialis
Pov: Perioval zone
Pove: Extratelencephalic component of the Pov
PRot: Perirotundic area
psp: Pallio-subpallial boundary
PT: Nucleus pretectalis
PTh: Prethalamus
PVN: Paraventricular nucleus of the hypothalamus
Rot: Nucleus rotundus
rp: Roof plate
Rtd: Dorsal reticular nucleus of the prethalamus
Rtv: Ventral reticular nucleus of the prethalamus
SbG: Subgeniculate nucleus
SCB: Suprachiasmatic hypothalamic band or domain
Se: Septum
SLu: Nucleus semilunaris
sod: Dorsal supraoptic decussation
SON: Supraoptic nucleus
SONdl: Dorsolateral SON
SONvm: Ventromedial SON
Sp: Subpallium
SPa: Subparaventricular nucleus of the hypothalamus
SpAr: Rostral division of the subpallial amygdalar area
SPO: Septopreoptic area
SPV: Supraopto-paraventricular hypothalamic domain
SPVt: Terminal prosomeric division of the SPV
SPVp: Peduncular prosomeric division of the SPV
St: Striatum or striatal embryonic division
StC: Striatal capsule (dorsal extension of the ITC-like patches)
Std: Dorsal division of St
TeO: Optic tectum
Th: Thalamus
TnA(P): Nucleus Taeniae (Pallial)
ToS: Torus semicircularis
Tu: Olfactory tubercle
Tup: Pallidal olfactory tubercle
vaf: Ventral amygdalofugal tract
VP: Ventral pallium
VPa: Ventral pallidum

## Footnotes

1 Enkephalin is known to modulate pain responses (König et al. 1996; Kang et al. 1998) and is also involved in stress-induced analgesia (Kung et al. 2010). pENK cells in the central amygdala are involved in inhibitory modulation of the activity of other EAce cells, including those projecting to pain control centers of the brainstem (such as the periaqueductal gray) (Haubensak et al. 2010; Li et al. 2013), and may be involved in stress-modulation of pain (Poulin et al. 2008). CRF is a well-known neuropeptide involved in stress and anxiety in mammals (Zorrilla and Koob 2004; Zorrilla et al. 2014), and CRF cells of the EAce are known to have descending projections to brainstem centers involved in autonomic and pain control (Moga and Gray 1985; Gray 1993), and have been involved in anxiety (Walker and Davis 2008). In mammals, SOM cells in the central amygdala appear to include two subpopulations: one coexpressing substance P (SP) and located in the medial subnucleus of the central amygdala (CeM), and another one without SP located in the lateral subnucleus (CeL) (Shimada et al. 1989). The latter was recently found to have direct projections to the brainstem and to be involved in learning and expression of fear conditioned responses (Li et al. 2013; Penzo et al 2014). Those in the CeM also appear to project to the brainstem (based on data on SP cells; Gray and Magnuson 1992), and—as typical of the CeM (Walker and Davis 2008)—may be involved in phasic fear responses (both conditioned and unconditioned) (see discussion in Vicario et al. 2015).
